# Temporal Dynamics and (Para)Clinical Factors Associated With (Long) Viral RNA Shedding in COVID‐19 Nonhospitalized Individuals – The COVID‐HOME Study

**DOI:** 10.1002/jmv.70125

**Published:** 2024-12-17

**Authors:** Larissa E. Vlaming‐van Eijk, Imran A. Ertugrul, Vinit Upasani, Karin I. Wold, María F. Vincenti‐Gonzalez, Alida C. M. Veloo, Arno R. Bourgonje, Daniele Pantano, Lilli Gard, Gerolf de Boer, Hubert G. M. Niesters, Alexander W. Friedrich, Marjolein Knoester, Bernardina T. F. van der Gun, Izabela A. Rodenhuis‐Zybert, Adriana Tami

**Affiliations:** ^1^ Department of Pathology and Medical Biology, Division of Pathology, University Medical Center Groningen University of Groningen Groningen The Netherlands; ^2^ Department of Cardiothoracic Surgery, University Medical Center Groningen University of Groningen Groningen The Netherlands; ^3^ Department of Medical Microbiology and Infection Prevention, University Medical Center Groningen University of Groningen Groningen The Netherlands; ^4^ Department of Gastroenterology and Hepatology, University Medical Center Groningen University of Groningen Groningen The Netherlands; ^5^ Department of Medicine, The Henry D. Janowitz Division of Gastroenterology Icahn School of Medicine at Mount Sinai New York USA; ^6^ University Hospital Münster Münster Germany

**Keywords:** COVID‐19, post‐COVID‐19 syndrome, SARS‐CoV‐2, viral shedding

## Abstract

Understanding temporal patterns and determinants of RNA shedding is important to comprehend SARS‐CoV‐2 transmission and improve biosafety/isolation guidelines. Nonhospitalized SARS‐CoV‐2‐infected individuals and household members were enrolled between March 2020 and June 2021 and followed prospectively ≥ 3 weeks during acute disease and at 3‐, 6‐, 12‐, and 18‐months to obtain (para)clinical data and biospecimens. Flow cytometry‐based surrogate assay (FlowSA) detected viable SARS‐CoV‐2. Determinants of long RNA shedding ( ≥ 21 days) were investigated. RNA shedding median duration was 14 days (IQR 8.0–21.0) for nasopharyngeal/throat (NPT) and 7 days (IQR 1.0–27.0) for feces— but 20 days (IQR 7.0–27.8) when excluding individuals positive at a single timepoint (25.2%). Among 17 NPT long shedders with FlowSA results, 12 (70.6%) demonstrated viable virus. NPT long shedding was independently positively associated with endocrine disease and chills. Fecal long shedding was independently inversely associated with age, female sex, and fatigue, but positively with vomiting. No associations with long‐term COVID‐19‐related complaints were observed. Finally, fecal long shedders demonstrated higher anti‐spike(S1) IgG levels over 18‐month follow‐up than non‐long shedders (*p* = 0.006). (Long) SARS‐CoV‐2 RNA shedding in NPT and feces associates with age and acute—but not prolonged—symptoms. The roles of prolonged infectious shedding and fecal shedding in transmission and immunity remain unclear.

## 1 Introduction

The emergence of severe acute respiratory syndrome coronavirus 2 (SARS‐CoV‐2), causing coronavirus disease 2019 (COVID‐19), rapidly disrupted healthcare and socioeconomic activities worldwide. Despite control measures, SARS‐CoV‐2 continues to circulate worldwide, albeit population‐based immunization and the evolution of favorable SARS‐CoV‐2 variants have contributed to attenuated disease burden. Still, challenges remain, including potential future emergence of new—more severe—SARS‐CoV‐2 variants and the condition in which people experience symptoms long after COVID‐19 diagnosis, known as the Post‐COVID‐19 syndrome (PCS). The persistence of SARS‐CoV‐2 RNA in various tissue compartments—either with replicating potential or not—alongside sustained inflammation has been hypothesized to be a potential explanation as to why patients experience lingering symptoms after infection [1, 2]. However, whether prolonged viral shedding ( ≥ 21 days) during the acute phase may contribute to developing PCS is currently unknown. Furthermore, the majority of studies rely solely on qRT‐PCR to determine viral shedding, but whether prolonged viral RNA shedding also includes viable virus remains elusive.

Prolonged shedding of SARS‐CoV‐2 RNA after acute infection has been reported in the literature, particularly in hospitalized individuals with comorbidities, older age, and those who receive immunosuppressive treatment [3, 4]. Nevertheless, studies on the duration of SARS‐CoV‐2 RNA shedding in nonhospitalized individuals with milder or asymptomatic COVID‐19 are still scarce, despite this group comprising the majority of infected individuals [5]. It is crucial to comprehend the temporal dynamics of viral shedding in COVID‐19 in order to improve the prevention of ongoing viral spread as well as to gain insight into risk factors for remaining COVID‐19‐related challenges, such as PCS. Here, we report longitudinal data on viral RNA shedding in multiple body specimen types from nonhospitalized SARS‐CoV‐2‐infected individuals. This study aims to gain insight in the temporal patterns and factors associated with (long) SARS‐CoV‐2 RNA shedding in individuals convalescing at home.

## 2 Methods

### 2.1 Study Population and Study Design

Nonhospitalized SARS‐CoV‐2‐infected individuals and their household members were enrolled between March 2020 and June 2021 in the COVID‐HOME study, an ongoing prospective longitudinal observational study in the Netherlands. The research protocol has been fully described elsewhere [6]. Briefly, a systematic weekly follow‐up was set up after the initial SARS‐CoV‐2‐infection to acquire data and samples to determine kinetics, length, viability, and routes of viral RNA shedding and their relationship with serological parameters and the patient's acute and long‐term clinical progression. Participants were followed up at 3‐, 6‐, 12‐, and 18‐months post‐enrolment to assess the presence of PCS and serological status. Study approval was obtained by the Medical Ethical Review Committee of the University Medical Center Groningen (UMCG) (METc no. 2020/158). Written informed consent was obtained from all participants. The study was conducted in accordance with the principles of the Declaration of Helsinki (2013) [7].

### 2.2 Data Collection and Sample Processing

Enrolled participants of all ages were visited at home within 48 h of initial diagnosis and then weekly on Days 7, 14, and 21 to obtain clinical data, a blood sample for serology determination, as well as nasopharyngeal/throat (NPT) swabs, feces and sperm/vaginal secretions (from consenting individuals > 16 years) for SARS‐CoV‐2 testing by qRT‐PCR [6]. SARS‐CoV‐2 infection was determined by qRT‐PCR positivity in either NPT or fecal samples. Cycle threshold (Ct) values were determined for all positive samples and values < 40 were considered positive. NPT swabs and fecal samples of noninfected household members were tested weekly to determine if and when each individual became qRT‐PCR positive. Patients who were positive after 3 weeks were invited to continue weekly sampling until negative. Duration of viral RNA shedding was defined as the number of days between the first and the last qRT‐PCR positive test result, after which results became negative. As not all participants were visited on the exact day of each weekly time point, a bidirectional range of 3 days was accepted (e.g., the Day 7 time point included visits between Days 4 and 10). Whole‐genome sequencing was performed on positive samples to determine the SARS‐CoV‐2 lineage. Blood samples were tested with the SARS‐CoV‐2 IgG II Quant Assay (Abbott) to determine IgG titers on Day 21 of acute disease and at 3‐, 6‐, 12‐, and 18‐months post‐enrollment. Serological results in AU/ml were converted into binding antibody units (BAU) by multiplying with a seroconversion factor = 0.142. Comorbidities were categorized as cardiovascular disease, pulmonary disease, endocrine disease, rheumatological disease, malignancy, neurological disease, and hematological disease. PCS was defined as a condition arising in individuals with a history of probable or confirmed SARS‐CoV‐2 infection, usually 3 months from the onset of COVID‐19, with symptoms that last for at least 2 months and cannot be explained by an alternative diagnosis [8].

### 2.3 Flow Cytometry‐Based Surrogate Assay (FlowSA) for the Detection of Viable Virus

As qRT‐PCR only detects total viral RNA load, a flow cytometry (FC)‐based surrogate assay (FlowSA) was used for the detection of viable SARS‐CoV‐2 particles in a pre‐selected sub‐population of 26 individuals. These 26 individuals were selected based on a duration of NPT viral RNA shedding > 14 days, Ct‐values < 40 and the availability of samples for analysis. Viability is defined here as the retention of the infective capacity of the virus in cell cultures (cell invasion and replication). The extensive methodology with validation of FlowSA for SARS‐CoV‐2 viable virus detection has been fully described elsewhere [9]. Briefly, an in‐house Flow‐SA was developed for detecting SARS‐CoV‐2 nucleocapsid (N) protein in Vero‐E6 cells exposed qRT‐PCR‐positive NPT samples from study participants. Incubation was done for 2 days and supernatants from these samples were harvested, cleared from cell debris and transferred to another batch of Vero‐E6 cells. These cells were intracellularly stained for the presence of SARS‐CoV‐2 N protein and subsequently detected and quantified using flow cytometry. Based on background staining for negative controls (infected Vero‐E6 cells stained only with fluorochrome‐conjugated secondary antibody), patient samples with ≤1% cells positive for SARS‐CoV‐2 were considered negative for FlowSA. Detection of SARS‐CoV‐2 N protein above the control levels indicates the presence of viable virus in patient NPT samples. A comparative analysis with conventional cytopathic effect (CPE)‐based cell culture assay was conducted to prove its sensitivity as a high‐throughput screening method of viable SARS‐CoV‐2 in NPT swabs [9].

### 2.4 Statistical Analysis

Duration of viral RNA shedding was analyzed as (a) a continuous outcome variable and (b) a binary outcome variable, where long shedding was defined as individuals with a duration of RNA shedding ≥ 21 days. Univariable and multivariable logistic regression analysis were performed to identify variables associated with prolonged SARS‐CoV‐2 RNA shedding. Multivariable analysis was performed using backward elimination (*P*_OUT_ > 0.05) and included all variables with a *p*‐value < 0.10 from univariable analysis. Continuous variables were converted into ordered categorical variables when suitable. Proportions were compared using Pearson's chi‐square tests and means and medians by Student's *t*‐test and Mann–Whitney *U*‐test, respectively. The Mantel–Haenszel score test examined trends in ordered categorical variables. Linear mixed models were used to assess changes in Ct‐values and anti‐S1 SARS‐CoV‐2 IgG antibody titers over time, in which time, sex, age group, specimen type (NPT or feces), as well as SARS‐CoV‐2 variant and lineage, were entered as fixed effects and subject as a random effect. Bonferroni correction was applied for multiple testing when appropriate. Anti‐S1 IgG antibody titers were log‐transformed prior to analysis. Estimated marginal means (EMM) were presented with corresponding standard deviations. Two‐tailed *p*‐values ≤ 0.05 were considered statistically significant. Data were analyzed anonymously using IBM SPSS Statistics Version 28.0 (IBM Corp. Armonk, NY, USA). Data visualization was realized using Adobe Illustrator (V.28.2), as well as Python programming language V3.9.7 (Python Software Foundation), using the *pandas* (V.1.3.3), *numpy* (V.1.21.2), *matplotlib* (V.3.4.3), *seaborn* (V.0.11.2), and *zepid* (V.0.9.1) packages.

## 3 Results

### 3.1 Cohort Characteristics

The total number of participants and samples tested, with proportions of positive results and duration of viral RNA shedding, are shown in Table 1. Among 256 enrolled participants, 190 (74.2%) tested positive for SARS‐CoV‐2 in any specimen at any time during the period of weekly sampling. Most participants provided NPT (245 [95.7%]) and fecal (225 [87.9%]) samples, which tested positive in 185 (75.5%) and 164 (72.9%) individuals, respectively. Amongst all females (*n* = 138), 87 (63.0%) provided vaginal secretions, which tested positive in 15 (17.2%) individuals. Furthermore, 48 (40.7%) of all males (*n* = 118) supplied semen samples with 8 (16.7%) participants testing positive. The median duration of SARS‐CoV‐2 RNA shedding was 14 days (IQR 8.0–21.0; range 1–43) for NPT and 7 days (IQR 1.0–27.0; range 1–41) for feces. Interestingly, 25.2% of individuals had a positive fecal sample at a single timepoint only, in contrast to 7.8% of individuals with a positive NPT sample at a single timepoint only (viral RNA shedding < 7 days) (Figure S1). When excluding these, the median duration in feces was 20 days (IQR 7.0–27.8), whereas in NPT, this was still 14 days (IQR 14.0–21.0).

**Table 1:** Participants and samples tested per specimen, including proportion of positive results and the duration of shedding in days.

	Total individuals tested (*n* = 256)	Total number of samples (mean number of samples per person)	Range number of samples per person	Positive individuals (%)	Median days of shedding (IQR)	Range shedding (days)
Any type of specimen	256 (100.0)	2322 (9.1)	0–7	190 (74.2)	14.0 (8.0–21.0)	1.0–43.0
NPT	245 (95.7)	1006 (4.1)	0–7	185 (75.5)	14.0 (8.0–21.0)	1.0–43.0
Feces						
*Total* *Positive* > *1 timepoint*	225 (87.9)	840 (3.7)	0–7	164 (72.9)	7.0 (1.0–27.0) 20.0 (7.0–27.8)	1.0–41.0 5.0‐41.0
Vaginal secretions (*n* = 138)^a^	87 (63.0)	321 (3.7)	0–5	15 (17.2)	NA^b^	NA^b^
Semen (*n* = 118)^c^	48 (40.7)	155 (3.2)	0–5	8 (16.7)	NA^b^	NA^b^

Abbreviations: IQR, interquartile range; NPT, nasopharyngeal/throat.

^a^ *n* = total number of female participants.

^b^ Due to missing data, no reliable duration of RNA shedding could be documented.

^c^ *n* = total number of male participants.

Baseline characteristics of participants with positive SARS‐CoV‐2 qRT‐PCR tests per specimen are shown in Table 2. Statistically significant differences in age group were observed between positive and negative SARS‐CoV‐2 individuals in both NPT (*p* = 0.039) and feces (*p* = 0.003). The age group 16–50 years tested positive more often (NPT: 56.8%, feces: 55.5%), followed by > 50 years (NPT: 30.3%, feces: 29.3%) and children ≤ 15 years (NPT: 13.0, feces: 15.2%). In both NPT and feces, the proportions of smoking status (NPT: *p* = 0.617, feces: *p* = 0.362), obesity (NPT: *p* = 0.231, feces: *p* = 0.098), and number of comorbidities (NPT: *p* = 0.143, feces: *p* =0.354) were comparable between individuals with positive and negative SARS‐CoV‐2 tests (Tables S1, S2).

**Table 2:** Baseline characteristics of subjects with positive SARS‐CoV‐2 qRT‐PCR test per specimen.

	NPT (*n* = 185) *n* (%)	Feces (*n* = 164) *n* (%)	Vaginal secretions (*n* = 15) *n* (%)	Semen (*n* = 8) *n* (%)
Age group				
≤ 15 16–50 > 50	24 (13.0) 105 (56.8) 56 (30.3)	25 (15.2) 91 (55.5) 48 (29.3)	—	—
Female sex	103 (55.7)	94 (57.3)	15 (100.0)	0 (0.0)
Smoking status Never Current Former	(*n* = 177) 130 (73.4) 9 (5.1) 38 (21.5)	(*n* = 159) 116 (73.0) 8 (5.0) 35 (22.0)	(*n* = 15) 12 (80.0) – 3 (20.0)	(*n* = 7) 6 (85.7) 1 (14.3) –
Obesity	(*n* = 137) 19 (13.9)	(*n* = 128) 19 (14.8)	(*n* = 13) 1 (7.7)	(*n* = 6) 0 (0.0)
Number of comorbidities 0 1 2–5	(*n* = 179) 121 (67.6) 34 (19.0) 24 (13.4)	(*n* = 161) 106 (65.8) 32 (19.9) 23 (14.3)	(*n* = 14) 11 (73.3) 2 (13.3) 2 (13.3)	(*n* = 8) 5 (62.5) 3 (37.5) –
Number of long shedders (≥ 21 days)	(*n* = 171)	(*n* = 132)		
	67 (39.2)	53 (40.2)	NA^a^	NA^a^

Abbreviation: NPT, nasopharyngeal/throat.

^a^ Due to missing data, no reliable number of long shedders could be given for vaginal secretions and semen.

According to the definition of PCS [8], the prevalence in the study population was 44.0% (55/125) at 3 months, 37.7% (49/130) at 6 months, 26.6% (34/128) at 12 months, 22.9% (19/83) at 18‐month follow‐up. Among SARS‐CoV‐2‐positive individuals, 50.9% (78/153) were infected with the Alpha variant, whereas the remaining 49.0% (75/153) were infected with pre‐Alpha lineages, including B.1.160 (*n* = 11), B.1.177 (*n* = 37), and B.1.221 (*n* = 14), among others. Out of 256 study participants, 138 (53.9%) reported receiving a COVID‐19 vaccination before or during the study, whereas the vaccination status of the remaining participants was unknown. The median time to receive the first vaccination dose after study enrollment was 77 days (IQR 52.0–140.0; range −82 to 359). However, only 8 (5.8%) of those that reported being vaccinated did so before enrollment or during the 3 weeks of acute disease.

### 3.2 Temporal Dynamics of Ct Values in Longitudinal NPT and Fecal Samples

**Figure 1:**
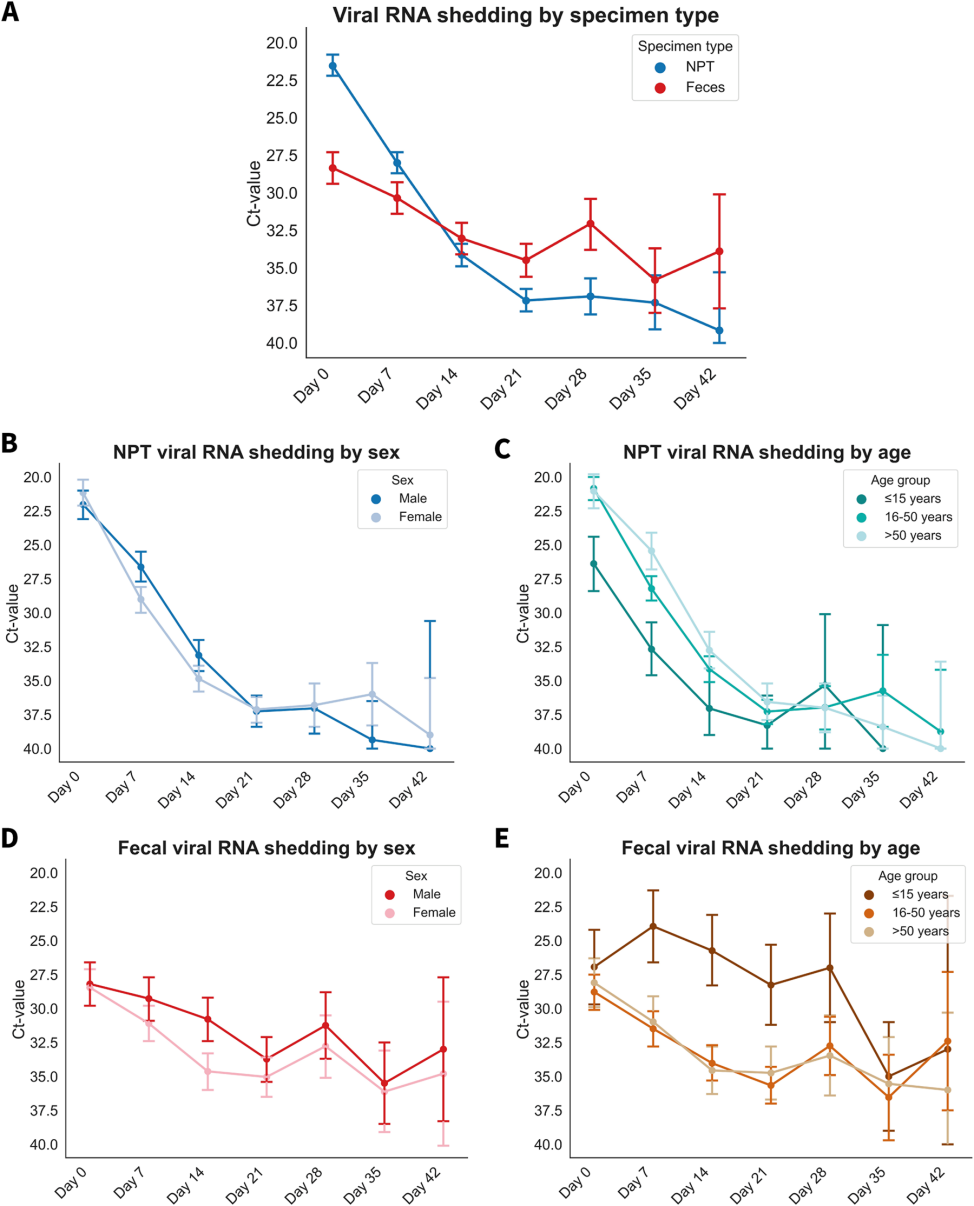
Temporal dynamics in Ct values in NPT and fecal samples by age and sex. (A) Viral RNA shedding by specimen type. (B) NPT viral RNA shedding by sex. (C) NPT viral RNA shedding by age. (D) Fecal viral RNA shedding by sex. (E) Fecal viral RNA shedding by age. Data are presented as estimated marginal means (EMM) of Ct values. The error bars represent the 95% confidence intervals. Abbreviation: NPT, nasopharyngeal/throat.

Figure 1 demonstrates the changes in Ct values over time in the acute phase for both NPT and fecal samples, stratified by sex and age. The Ct values in both NPT and fecal samples significantly changed over time during the acute phase (up to Day 42) (*p* < 0.001). The Ct values in NPT swabs showed a steep incline during the first 3 weeks after SARS‐CoV‐2 infection and kept declining up to 7 weeks, whereas Ct values remained more stable over time during at least 7 weeks in fecal samples as compared to NPT samples, suggesting longer SARS‐CoV‐2 RNA shedding in feces (Figure 1A). When comparing EMM over time during the acute phase, significantly lower Ct values were observed in males (EMM ± SD 30.9 ± 0.4) as compared to females (32.5 ± 0.4) (*p* = 0.002) in feces (Figure 1D, Table S3), whereas no noticeable differences were observed between males (30.4 ± 0.4) and females (31.2 ± 0.4) in NPT swabs (*p* = 0.140) (Figure 1B, Table S4). When stratifying by age group, the age group ≤ 15 years (33.5 ± 0.8) demonstrated significantly higher Ct values as compared to age groups 16–50 years (30.7 ± 0.4) (*p* = 0.002) and > 50 years (30.1 ± 0.5) (*p* < 0.001) in NPT swabs (Figure 1C, Table S4). However, as for feces, lower Ct values were observed in age group ≤ 15 (27.1 ± 0.7) when compared to age groups 16–50 years (32.6 ± 0.3) (*p* < 0.001) and > 50 years (32.4 ± 0.5) (*p* < 0.001) (Figure 1E, Table S3). In NPT, significant differences were observed in Ct values over time when comparing the Alpha surge (29.8 ± 0.4) with the pre‐Alpha surge (31.2 ± 0.4) (*p* = 0.029) (Figure S2A and Table S4). When further stratifying by lineage, significantly higher Ct values were observed in individuals infected by the B.1.160 strain (32.2 ± 1.1) as compared to the B.1.1.7/Alpha strain (29.8 ± 0.4) (*p* = 0.047) (Figure S2B and Table S4). No significant differences were observed in Ct values over time between Alpha vs. pre‐Alpha surges and SARS‐CoV‐2 lineages in feces (Figure S2C, D and Table S3).

### 3.3 The Duration of Viral RNA Shedding Differs per age Group but not per SARS‐CoV‐2 Variant

**Figure 2:**
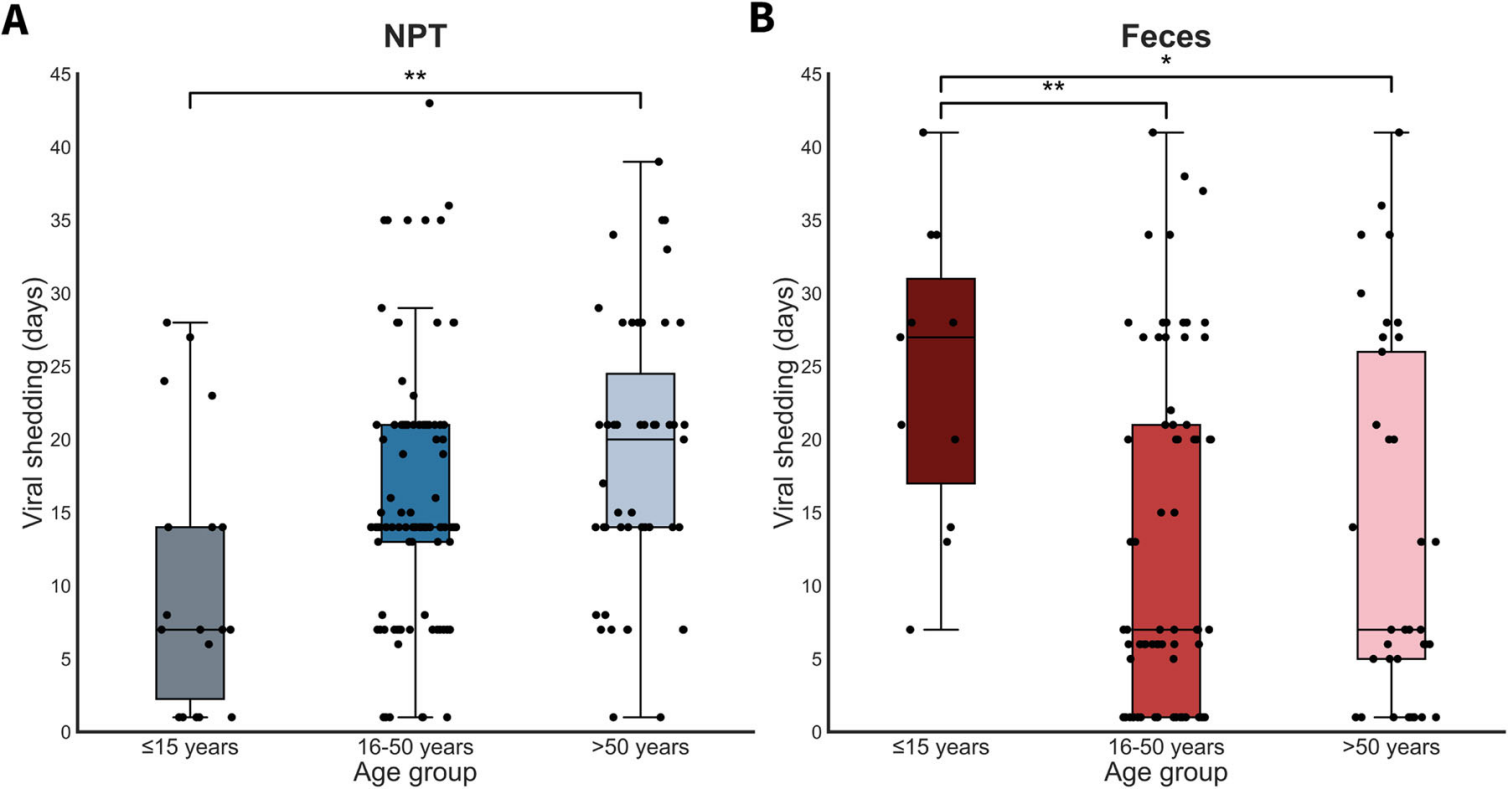
Duration of viral RNA shedding (in days) by age group. (A) NPT viral RNA shedding increases with age, with a significant difference between age groups ≤ 15 and > 50 years (*p* = 0.004). (B) Fecal viral RNA shedding decreases with age, with significant differences between age groups ≤ 15 and 16–50 years (*p* = 0.005), and between age groups ≤ 15 and > 50 years (*p* = 0.024). Each point denotes the maximum duration of viral RNA shedding per individual.

The duration of viral RNA shedding increased with age, with a significant difference between individuals ≤ 15 years and those > 50 years (*p* = 0.004) (Figure 2A). In contrast, the total days of viral RNA shedding in feces decreased with age, with longer shedding for individuals aged ≤ 15 compared to those aged 16–50 years (*p* = 0.005) and > 50 years (*p* = 0.024) (Figure 2B). The duration of viral RNA shedding in NPT and feces did not differ per SARS‐CoV‐2 variant (pre‐Alpha vs. Alpha) or lineage (Figure S3).

### 3.4 Associations Between Clinical Parameters and the Odds of Long Viral RNA Shedding

**Figure 3:**
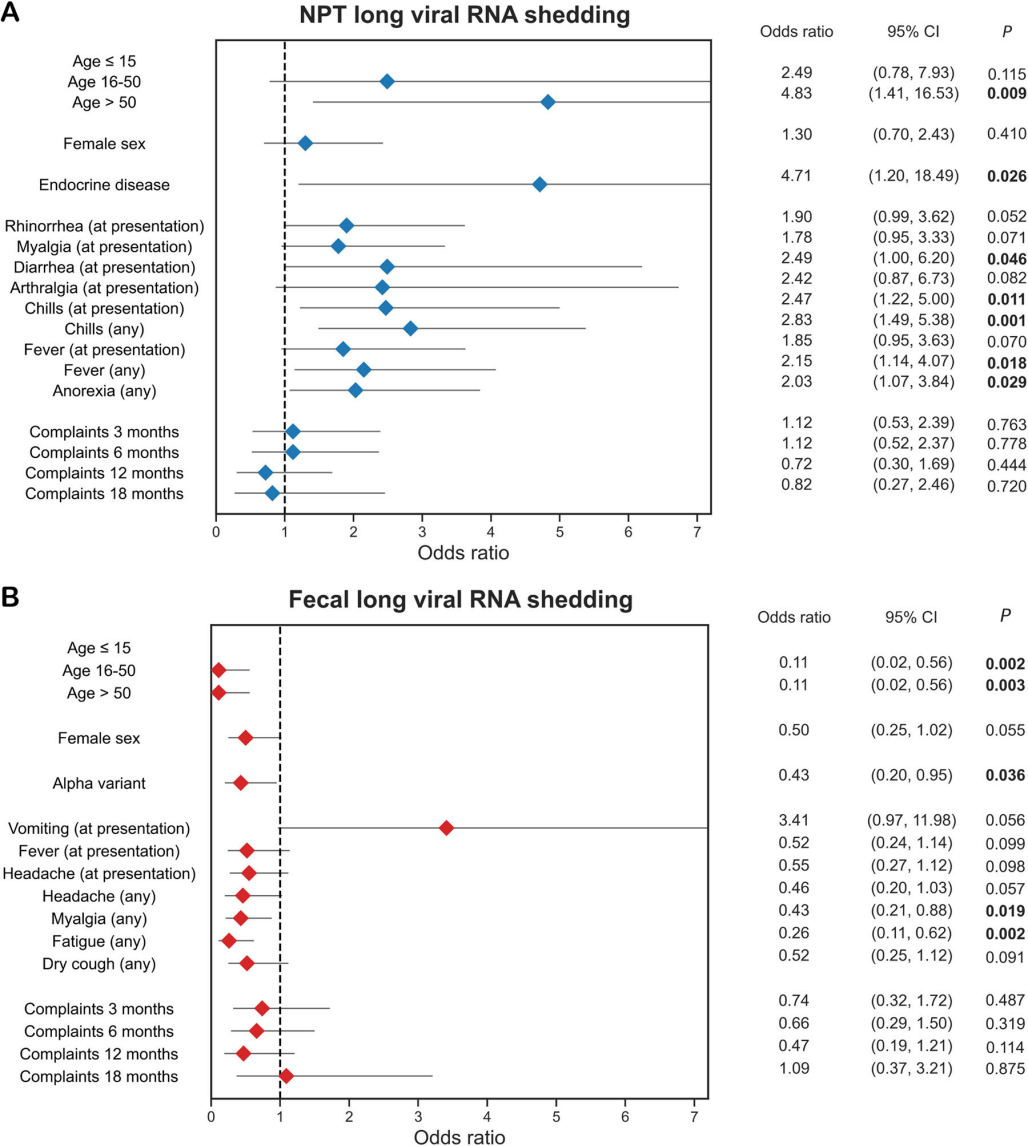
Univariable analysis of association between clinical parameters and the odds of viral long shedding. (A) The likelihood of NPT long shedding (≥ 21 days) increases with age (chi‐square test for trend *p* = 0.006) and positively associates with diarrhea and chills as presenting symptom, and with chills, fever, and anorexia at any time during the 21‐day follow‐up. (B) The likelihood of long shedding in feces (≥ 21 days) decreases with age (chi‐square test for trend *p* = 0.030) and inversely associates with myalgia and fatigue at any time during the 21‐day follow‐up. Abbreviations: NPT, nasopharyngeal/throat; CI, confidence intervals.

To identify clinical parameters that were associated with prolonged viral RNA shedding in NPT and fecal samples, univariable and multivariable logistic regression analyses were performed (Tables S5, S6). We found that long RNA shedding (defined as ≥ 21 days) in NPT swabs occurred in 39.2% (67/171) of individuals and in 40.2% (53/132) of individual's fecal samples. Univariable logistic regression showed that the likelihood of long RNA shedding in NPT swabs (defined as ≥ 21 days) increased with age (*p* = 0.006) but did not differ by sex (*p* = 0.410) (Figure 3A). Long SARS‐CoV‐2 RNA shedding in NPT was positively associated with having diarrhea (OR = 2.49, *p* = 0.046) and chills (OR = 2.47, *p* = 0.011) at presentation, and with chills (OR = 2.83, *p* = .001), fever (OR = 2.15, *p* = 0.018), and anorexia (OR = 2.03, *p* = 0.029) at any time during the 21‐day follow‐up. No associations were observed between NPT long RNA shedding and smoking, obesity or number of comorbidities, nor with SARS‐CoV‐2 variant (pre‐Alpha vs. Alpha) or lineage. When investigating the associations with type of comorbidity, only endocrine disease showed a significant association with NPT long RNA shedding (OR = 4.71, *p* = 0.026). In multivariable logistic regression analysis, only endocrine disease (OR = 4.29, *p* = 0.042) and chills at any time during the 21‐day follow‐up (OR = 2.76, *p* = 0.003) remained statistically significant.

The likelihood of long shedding in feces ( ≥ 21 days) decreased with age (*p* = 0.030) and was more likely to occur in males (*p* = 0.055) (Figure 3B) in univariable logistic regression analysis. An inverse association was observed between fecal long shedding and myalgia (OR = 0.43, *p* = 0.019) and fatigue (OR = 0.26, *p* = 0.002) at any time during the 21‐day follow‐up, as well as with Alpha SARS‐CoV‐2 variant (OR 0.43, *p* = 0.036). No associations were observed between fecal long RNA shedding and smoking, obesity or type of comorbidities. Having one comorbidity was associated with a decreased likelihood of long RNA shedding in feces as compared to no comorbidities (OR = 0.21, *p* = 0.008), although trend analysis was not statistically significant (*p* = 0.161). Multivariable logistic regression analysis showed that an age 15–50 years (OR = 0.12, *p* = 0.015), age > 50 years (OR = 0.09, *p* = 0.009), female sex (OR = 0.39, *p* = 0.025), vomiting at presentation (OR = 5.31, *p* = 0.020) and fatigue at any time during the 21‐day follow‐up (OR = 0.29, *p* = 0.011) were independently associated with fecal long RNA shedding. No associations were observed between NPT nor fecal long RNA shedding and the presence of PCS complaints during long‐term follow‐up.

### 3.5 Long RNA Shedding in Feces Associates With anti‐S1 SARS‐CoV‐2 IgG During Long‐Term Follow‐Up

**Figure 4:**
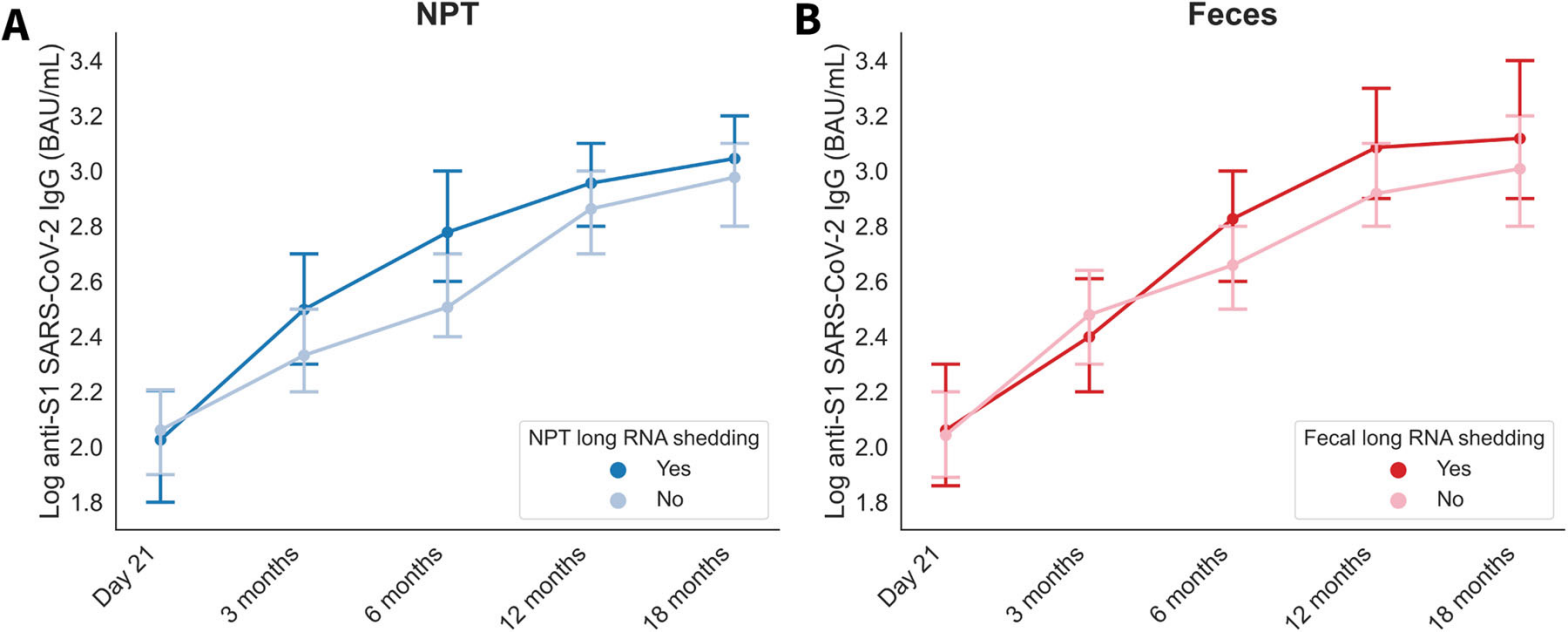
Comparison of anti‐S1 SARS‐CoV‐2 IgG levels in individuals with and without long viral RNA shedding. (A) NPT. (B) Feces. Data are presented as estimated marginal means (EMM) of log‐transformed anti‐S1 SARS‐CoV‐2 IgG levels in BAU/mL. The error bars represent 95% confidence intervals. Abbreviations: D, days; M, months; NPT, nasopharyngeal/throat.

To examine whether long RNA shedding associates with post‐infection IgG antibody response, log‐transformed anti‐S1 IgG levels over time were compared between long‐shedders and non‐long‐shedders in both NPT and feces using linear mixed model analyses (Figure 4, Tables S7, S8). Individuals with long fecal RNA shedding had significantly higher anti‐S1 IgG levels (EMM ± SD 2.71 ± 0.05) during the 18‐month follow‐up as compared to non‐long fecal RNA shedders (2.54 ± 0.04) (*p* = 0.006). Similarly, higher anti‐S1 IgG levels were observed in NPT long shedders (2.65 ± 0.05) as compared to non‐long shedders (2.53 ± 0.04), with a statistical difference close to significance (*p* = 0.056).

### 3.6 Viable Viral Shedding in NPT can Last 21 Days or Longer

**Figure 5:**
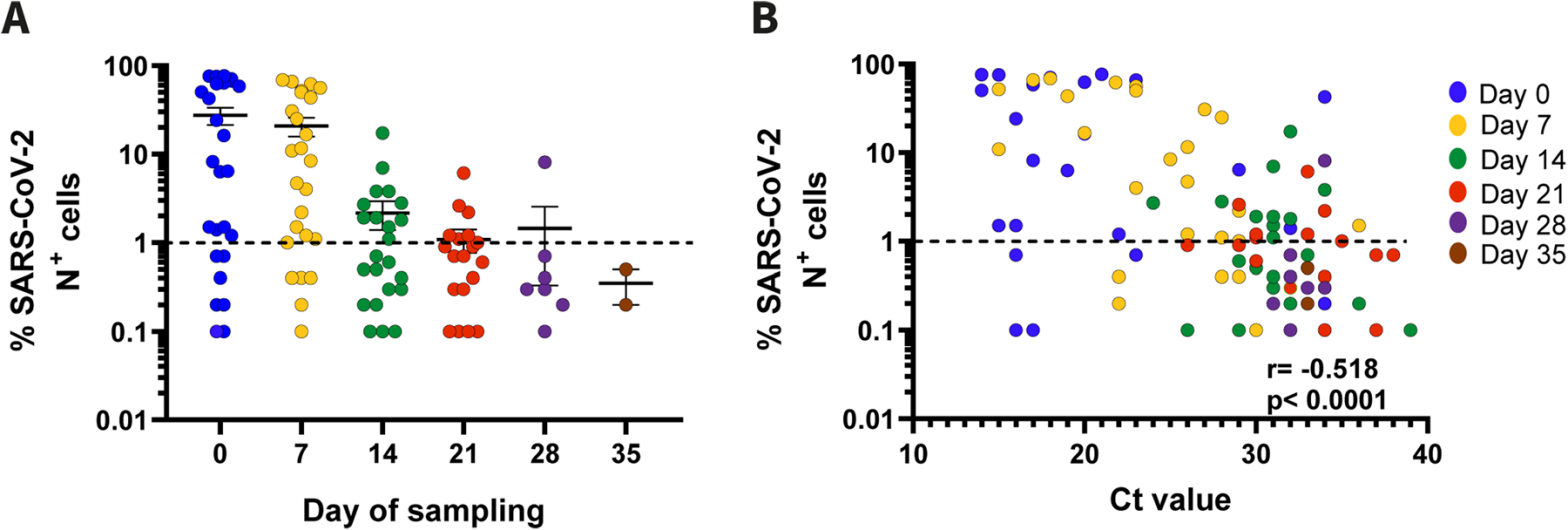
Detection of viable virus in selected samples by sampling day and Ct value. (A) Detection levels of N protein in NPT samples by FlowSA methodology, stratified by day of sampling. Error bars represent mean ± standard error of the mean (SEM). Dotted line indicates threshold below which NPT samples are considered negative for FlowSA. (B) Correlation between FlowSA results and Ct values for SARS‐CoV‐2 E gene of respective Association of percentage of SARS‐CoV‐2 N^+^ cells with SARS‐CoV‐2 qRT‐PCR Ct values were determined using Spearman's correlation method.

Among the 26 individuals in whom FlowSA was performed, viable virus was mostly detected in samples from Day 0 to Day 14, although a steep decline was observed after Day 7 (Figure 5A). Still, 60% of individuals with available samples remained positive for viable SARS‐CoV‐2 until Day 21. Furthermore, the percentages of SARS‐CoV‐2 N+ cells obtained with the NPT samples inversely correlated with their corresponding Ct values of SARS‐CoV‐2 PCR (*p* < 0.0001; Figure 5B). Altogether, these results indicate that FlowSA detected viable SARS‐CoV‐2 in NPT swabs with high Ct values up to 39. Out of 17 individuals with long RNA shedding in NPT swabs in which FlowSA was performed, 13 (76.5%) individuals showed the presence of viable virus in NPT samples > 14 days, and 12 (70.6%) demonstrated viable virus in NPT samples > 21 days.

## 4 Discussion

In this study, we present data on viral RNA shedding temporal dynamics, routes, duration, and associated factors. We reported a median viral RNA shedding duration of 14 days (IQR 8.0–21.0; range 1–43) in NPT samples and 7 days (1.0–27.0; range 1–41) for fecal ones. However, a longer median duration of 20 days (IQR 7.0–27.8) in feces was observed when excluding the 25.2% of individuals that were positive at a single time point only. Using a novel FlowSA, we showed that most individuals with NPT long RNA shedding had detectable viable virus in samples > 14 days (13/17, 76.5%) and > 21 days (12/17, 70.6%). In addition, we demonstrated that the likelihood of long NPT shedding (> 21 days) increased with age, whereas long shedding in feces decreased with age. Additional associations were found between long RNA shedding and endocrine disease, as well as clinical symptoms, with positive associations between NPT long shedding and diarrhea and chills at presentation, and with chills, fever, and anorexia at any time during the 21‐day follow‐up. After adjustment for confounding factors, the associations with endocrine disease and chills during the 21‐day follow‐up remained statistically significant. Fecal long shedding was significantly negatively associated with myalgia and fatigue at any time during the 21‐day follow‐up. After adjustment for confounding factors, fecal long shedding appeared to be independently associated with age, sex, vomiting at presentation, and fatigue at any time during the 21‐day follow‐up. Interestingly, no significant associations were observed between long RNA shedding and the presence of prolonged COVID‐19 complaints. Finally, we reported higher anti‐S1 IgG levels over long‐term follow‐up in long RNA shedders as compared to non‐long RNA shedders in both NPT and feces, albeit this difference only reached statistical significance in feces.

Overall, Ct values in NPT and feces inclined over time during the acute phase, with Ct values remaining more stable in feces during at least 7 weeks as compared to NPT, suggesting longer viral RNA shedding in feces. Indeed, after excluding those cases with SARS‐CoV‐2 positivity at a single timepoint only, we found a longer median duration of viral RNA shedding in feces as compared to NPT, supporting findings from others [10, 11]. Hence, relatively many individuals with positive fecal sampling shed for a very short time (< 7 days), but if they did shed longer, then this is substantially longer than that from NPT viral RNA shedding.

Our findings suggest that NPT viral RNA shedding increases with age, whereas fecal viral RNA shedding decreases with age and is predominantly found in children, both reflected by Ct values and the duration of viral positivity. Similar findings that fecal‐oral transmission could be a prominent transmission route in children have also been observed by others [12, 13, 14, 15]. Prolonged SARS‐CoV‐2 shedding and more severe illness in the elderly are thought to (at least partly) result from a weakened immune response, leading to increased viral replication with resulting higher and prolonged viral load [16]. Existing comorbidities in the elderly may also impact viral shedding through various ways—such as by changes in ACE2 receptor and immune‐mediating cytokines—leading to prolonged and more severe illness in this population [16]. Several potential mechanisms may be involved in the longer‐term fecal viral shedding in children as compared to adults, including differences in the gut microbiota, mucosal immune system, and/or expression of SARS‐CoV‐2‐related entry factors in the gastrointestinal tract, as well as poorer hand hygiene practices and higher tendency of silent aspiration of sputum [13].

Sex as associate of SARS‐CoV‐2 RNA shedding has been reported by others with conflicting results [17, 18, 19]. In this study, males demonstrated significantly lower Ct values in feces over time, as well as an increased likelihood of being a long shedder in feces, even after adjustment for relevant confounding factors. However, no significant differences were found in Ct values and prolonged NPT viral RNA shedding by sex.

We found higher anti‐S1 IgG antibody titers in NPT and fecal viral long shedders as compared to non‐long shedders over the long term. These findings suggest that prolonged viral load during the acute phase of infection may promote antibody production over time, and that long shedders may be better protected against reinfection since lower IgG levels have been associated with an increased risk of re‐detectable viral RNA [20].

In our study, we identified noteworthy correlations between certain symptoms with prolonged NPT and fecal shedding, including independent associations between prolonged NPT shedding and chills at 21‐day follow‐up, as well as between prolonged fecal shedding and vomiting at presentation and fatigue at 21‐day follow‐up, suggesting that the clinical course of COVID‐19 may, to some extent, influence the duration of viral RNA shedding. However, no associations were observed between prolonged viral RNA shedding in NPT and feces and experiencing complaints at the 3‐, 6‐, 12‐, and 18‐month follow‐up. Hence, our findings suggest that prolonged SARS‐CoV‐2 shedding during the acute phase does not contribute to developing PCS. PCS is complex and thought to occur from a convoluted interplay of multiple pathophysiological mechanisms, including immune dysregulation, autoimmunity, endothelial dysfunction with coagulation activation, changes to the gut microbiota, and occult viral persistence, among others [1].

The ongoing evolution of SARS‐CoV‐2 has resulted in the emergence of numerous variants, which have shown differences in viral load and the duration of viral shedding compared to the original SARS‐CoV‐2 strain [21]. In this study, conducted during the first year and a half of the pandemic, about half of the participants were infected with the Alpha variant, while the remaining participants were infected with various pre‐Alpha lineages, including B.1.160, B.1.177, and B.1.221. For NPT samples, Alpha infection was associated with lower Ct values over time during acute infection, consistent with other research showing a higher RNA viral load compared to the ancestral strain [22]. In our study, there were no observed differences in the median durations of viral RNA shedding between variants or lineages in NPT or feces, but Alpha was significantly inversely associated with being a long shedder in feces. It is essential to acknowledge that pre‐existing immunity to SARS‐CoV‐2, whether from previous infection or vaccination, could have driven these differences in viral load and viral shedding duration. Indeed, in our study, over half of the participants were known to have been vaccinated, with a median of 77 days after study enrollment, which may have influenced our follow‐up data to some degree. Of note, the range was from –82 to 359 days, indicating that some participants received the vaccine even before study enrollment. However, only 8 participants (5.8% of those who reported being vaccinated) received the vaccine either before study enrollment or during the first 3 weeks of acute disease, potentially influencing their long‐shedding results. The remaining 94.2% were vaccinated later, well after the acute disease phase, and thus their vaccination status would not have affected the long shedding (> 21 days) outcomes in our study.

The current SARS‐CoV‐2 variants may show a different viral shedding pattern compared to our findings. A recent systematic review provided an overview of current evidence on the daily SARS‐CoV‐2 culture positivity rates including the delta and omicron variants [23]. They observed that culture positivity was 44% to 50% on Days −1 to 5, followed by a decline to 28% at Day 7% and 11% at Day 9, and 0% beyond Day 18 since symptom onset or diagnosis [23]. Overall, the duration of RNA and viable viral shedding appears to have decreased with the evolution of SARS‐CoV‐2 variants, although it should be noted that pre‐existing infection‐induced or vaccination‐induced immunity may influence these differences in shedding duration [21, 23]. Current data on fecal viral shedding is limited. However, *Wannigama* et al. recently confirmed the differences in viral shedding rates between SARS‐CoV‐2 variants, with fecal viral shedding prevalence up to 21 days after symptom onset, which is in line with our results [24]. The current viral shedding duration, however, will generally depend on factors such as the severity of the illness, vaccination status, and immune response.

In this study, viral RNA was detected using qRT‐PCR, which is currently the most widely used diagnostic method for SARS‐CoV‐2 detection owing to its reliability and sensitivity [25, 26, 27]. However, as qRT‐PCR is not appropriate for discriminating between total viral load and viable virus particles, additional FlowSA was used to determine the presence of viable virus in qRT‐PCR‐positive NPT samples from study participants. Indeed, most individuals with long RNA shedding in NPT also showed the presence of viable virus particles, both in samples > 14 days and > 21 days, implying that these individuals were still capable of producing infectious virus particles beyond the initially agreed isolation time of 7–14 days [28]. However, it must be taken into consideration that the titers of infectious viral particles in the nasopharyngeal discharge of these individuals, with very high qRT‐PCR Ct values (> 39), may not be sufficient to sustain further viral transmission to other individuals. Since FlowSA was performed in a small subset (*n* = 26) of individuals, event rates were too low to reliably assess clinical parameters associated with viable viral shedding.

Quarantine requirements varied by country and period, with guidelines differing based on factors such as exposure risk, the presence of symptoms, and the availability of testing. In the Netherlands, quarantine guidelines evolved over time, initially requiring symptomatic individuals to isolate for a minimum of 7 days (provided they were symptom‐free for at least 24 h) and a maximum of 14 days, which was later adjusted to 5–10 days when the Omicron variant became dominant [29, 30]. The initial isolation time of 14 days is in line with our results of a median RNA shedding time in NPT of > 14 days. However, given that a notable proportion (39.2%) of individuals were long RNA shedders in NPT and that viable virus could be detected beyond 14 and 21 days making them potentially contagious, it would have been beneficial to adjust the isolation recommendations as a biosecurity measure for individuals with prolonged NPT RNA shedding. At the time, however, direct identification of long RNA shedders was challenging, since national testing only became available in June 2020, after which testing capacity remained limited, and the decision to end quarantine was based on the recommended isolation duration and symptom resolution rather than a negative test result [29, 30]. Our study has identified factors associated with prolonged viral RNA shedding, for which additional measures may be proposed. Specifically, we recommend that individuals of older age (> 50 years) should take additional preventive measures to avoid spreading the virus, such as masking, washing hands frequently, and/or isolating for at least 14 days. Furthermore, a larger, more comprehensive analysis of individuals with viable virus would be valuable in understanding how long infectious virus is shed, particularly through the respiratory tract. This could help refine isolation guidelines and improve risk assessment strategies for those with prolonged infectious shedding. Finally, our study highlights that viral shedding in stools can persist for longer than in NPT, which may indicate an ongoing risk of transmission through fecal‐oral routes. Furthermore, the prolonged RNA shedding in fecal matter may be associated with other clinical outcomes, such as alterations in the gut microbiota [31, 32].

Strengths of this study include its prospective design, following individuals from the day of infection, facilitating the analysis of associations between viral RNA shedding and clinical parameters. The presence of sequential measurements over time allowed us to gain insight into the dynamics of Ct values in NPT and feces, and their associations with relevant clinical and laboratory parameters during the acute and long‐term disease course. In addition, we also investigated the presence of viable virus particles to understand the duration of infectious SARS‐CoV‐2, a relevant parameter for biosafety guidelines. Several limitations to this study warrant recognition. First, this study was conducted during the first three waves of the pandemic with results reflecting a mainly non‐vaccinated population infected with SARS‐CoV‐2 variants dominating that period. Therefore, our findings may not necessarily be translatable to the present situation with a mostly vaccinated population in which Omicron subvariants are dominating. Furthermore, because of the longitudinal study design, different factors during study participation could have affected our long‐term data, with vaccination and potential SARS‐CoV‐2 reinfection being the most noteworthy factors. Finally, some of the predesigned sampling was missing, which may have caused bias in determining the viral RNA shedding duration in days, although this did not influence categorizing individuals in long shedders and non‐long shedders based on the cut‐off of 21 days, and therefore had no effect on the binary outcome of long shedding.

In conclusion, we here demonstrated the temporal dynamics of viral shedding in nonhospitalized COVID‐19 individuals convalescing at home. Long viral RNA shedding in NPT and feces was observed, which appeared to be associated with age (depending on sample specimen), endocrine disease (NPT), sex and SARS‐CoV‐2 variant (feces), COVID‐19‐related clinical symptoms, and postinfection IgG antibody titers, but not with prolonged COVID‐19 complaints. Our results add to the currently incomplete understanding of SARS‐CoV‐2 RNA shedding, which is vital in the prevention of ongoing viral spread and new outbreaks with the potential emergence of new SARS‐CoV‐2 variants.

## Author Contributions

**Larissa E. Vlaming‐van Eijk:** data curation, formal analysis, visualization, writing–original draft, writing–review and editing. **Imran A. Ertugrul:** data curation, formal analysis, writing–original draft, writing–review and editing. **Vinit Upasani:** data curation, formal analysis, visualization, writing–review and editing. **Karin I. Wold:** data curation, investigation, project administration, writing–review and editing. **María F. Vincenti‐Gonzalez:** data curation, investigation, methodology, formal analysis, writing–review and editing. **Alida C. M. Veloo:** data curation, investigation, writing–review and editing. **Arno R. Bourgonje:** formal analysis, visualization, writing–review and editing. **Daniele Pantano:** data curation, investigation, methodology, project administration, writing–review and editing. **Lilli Gard:** data curation, investigation, methodology, validation, writing–review and editing. **Gerolf de Boer:** data curation, software, writing–review and editing. **Alex W. Friedrich:** funding acquisition, investigation, project administration, supervision, writing–review and editing. **Marjolein Knoester:** funding acquisition, data curation, investigation, methodology, writing–review and editing. **Bernardina T. F. van der Gun:** funding acquisition, data curation, Investigation, project administration, supervision, writing–review and editing. **Izabela A. Rodenhuis‐Zybert:** conceptualization, investigation, methodology, supervision, writing–review and editing. **Adriana Tami:** conceptualization, data curation, funding acquisition, investigation, methodology, project administration, supervision, writing–original draft, writing–review and editing. All authors have read and agreed to the published version of the manuscript.

## Conflicts of Interest

The authors declare no conflicts of interest.

## Supporting information

Supporting information.

Supporting information.

Supporting information.

Supporting information.

## Data Availability

The data that support the findings of this study are available from the corresponding author upon reasonable request. Deidentified data will be available upon written request from the corresponding author.
