# Supplementary material for: Temporal Dynamics and (Para)Clinical Factors Associated With (Long) Viral RNA Shedding in COVID‐19 Nonhospitalized Individuals – The COVID‐HOME Study
Source: J Med Virol. 2024 Dec 17;96(12):e70125. doi: 10.1002/jmv.70125 (PMC11653057; doi:10.1002/jmv.70125)
Supplement: Supplementary file 1 — Supporting information. [file JMV-96-e70125-s004.docx]

**Supplementary Materials**

**
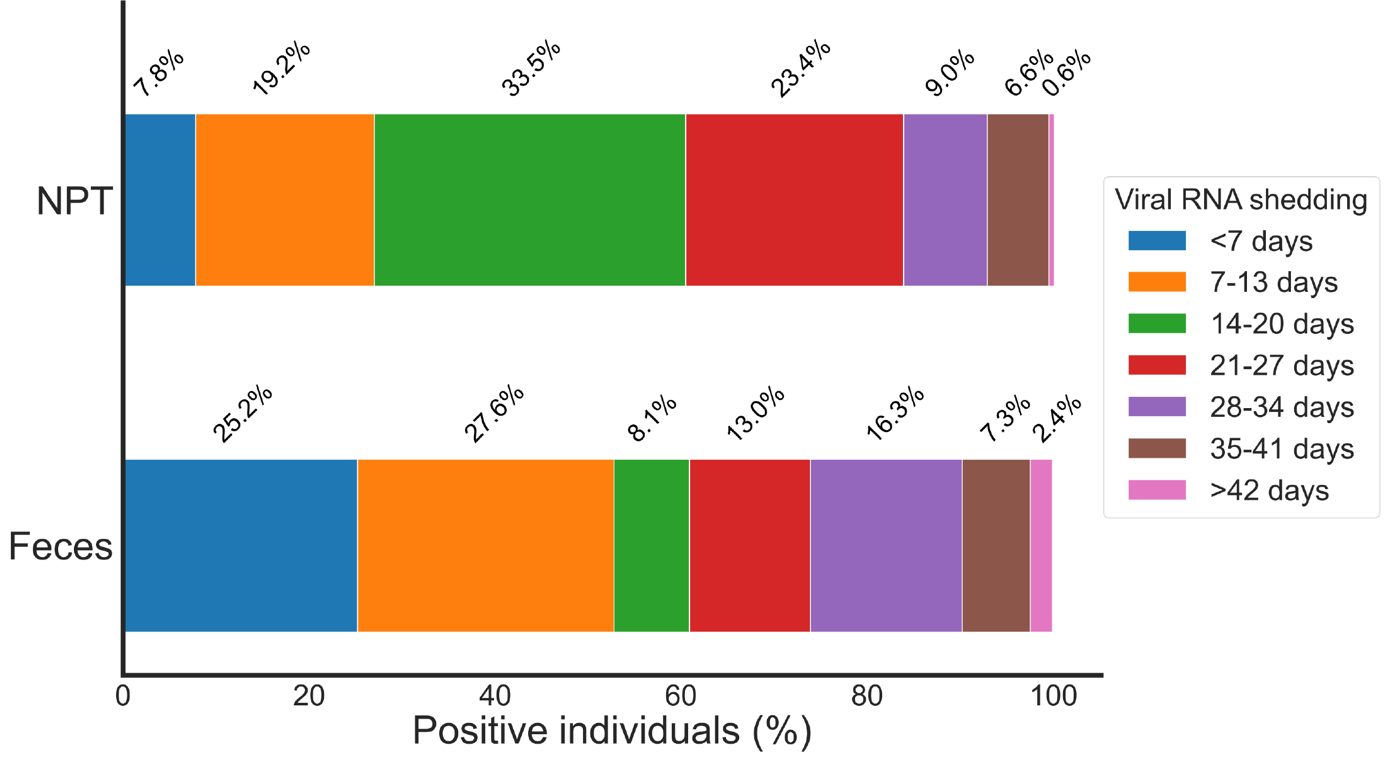
**

**Supplementary Figure S1. Percentages of positive samples per amount of time points in NPT and feces.** Each colored bar represents the proportion of individuals testing positive in NPT and feces per amount of time points. Blue = positive at 1 time point only (RNA shedding <7 days); Orange = positive at 2 time points (RNA shedding 7-13 days); Green = positive at 3 time points (RNA shedding 14-20 days); Red = positive at 4 time points (RNA shedding 21-27 days); Purple = positive at 5 time points (RNA shedding 28-34 days); Brown = positive at 6 time points (RNA shedding 35-41 days); Pink = positive at ≥6 time points (RNA shedding >42 days). As not all participants were visited on the exact day of each weekly time point, a bidirectional range of 3 days per each time point was accepted. Abbreviations: NPT, nasopharyngeal/throat.

**Supplementary Table S1. Baseline characteristics of subjects with positive and negative SARS-CoV-2 qRT-PCR in nasopharyngeal/throat (NPT) samples.**

|  | **Positive qRT-PCR**  ***n* (%)** | **Negative qRT-PCR**  ***n* (%)** | ***p*-value** |
| --- | --- | --- | --- |
| Age group  ≤ 15  16-50  > 50 | 24 (13.0)  105 (56.8)  56 (30.3) | 15 (25.0)  34 (56.7)  11 (18.3) | **0.039** |
| Female sex | 103 (55.7) | 28 (46.7) | 0.224 |
| Smoking status  Never  Current  Former | 130 (73.4)  9 (5.1)  38 (21.5) | 13 (68.4)  2 (10.5)  4 (21.1) | 0.617 |
| Obesity | 19 (13.9) | 0 (0.0) | 0.231 |
| Number of comorbidities  0  1  2-5 | 121 (67.6)  34 (19.0)  24 (13.4) | 13 (68.4)  6 (31.6)  0 (0.0) | 0.143 |

Abbreviations: RT-PCR, reverse transcriptase-polymerase chain reaction.

**Supplementary Table S2. Baseline characteristics of subjects with positive and negative SARS-CoV-2 qRT-PCR in feces.**

|  | **Positive qRT-PCR**  ***n* (%)** | **Negative qRT-PCR**  ***n* (%)** | ***p*-value** |
| --- | --- | --- | --- |
| Age group  ≤ 15  16-50  > 50 | 25 (15.2)  91 (55.5)  48 (29.3) | 21 (34.4)  31 (50.8)  9 (14.8) | **0.003** |
| Female sex | 94 (57.3) | 29 (47.5) | 0.190 |
| Smoking status  Never  Current  Former | 116 (73.0)  8 (5.0)  35 (22.0) | 16 (64.0)  3 (12.0)  6 (24.0) | 0.362 |
| Obesity | 19 (14.8) | 0 (0.0) | 0.098 |
| Number of comorbidities  0  1  2-5 | 106 (65.8)  32 (19.9)  23 (14.3) | 18 (72.0)  6 (24.0)  1 (4.0) | 0.354 |

Abbreviations: RT-PCR, reverse transcriptase-polymerase chain reaction.

**Supplementary Table S3. Results of linear mixed models of Ct values in feces.**

|  | **Estimated marginal means (EMM)** | **SE** | **95% CI** | ***p*-value*** | **F-statistic** | ***p*-value** |
| --- | --- | --- | --- | --- | --- | --- |
| **Timepoint**  T0 (Day 0)  T1 (Day 7)  T2 (Day 14)  T3 (Day 21)  T4 (Day 28)  T5 (Day 35)  T6 (Day 42) | 28.35  30.35  33.04  34.49  32.06  35.81  33.90 | 0.53  0.53  0.53  0.56  0.87  1.09  1.95 | 27.3-29.4  29.3-31.4  32.0-34.1  33.4-35.6  30.4-33.8  33.7-38.0  30.1-37.7 | -  **0.008**  **<0.001**  **<0.001**  **<0.001**  **<0.001**  **0.006** | 15.604 | **<0.001** |
| **Sex**  Female  Male | 32.48  30.85 | 0.35  0.40 | 31.8-33.2  30.1-31.6 | **0.002**  - | 9.393 | **0.002** |
| **Age group (years)**  ≤15  16-50  >50 | 27.11  32.60  32.43 | 0.69  0.34  0.47 | 25.8-28.5  31.9-33.3  31.5-33.3 | -  **<0.001**  **<0.001** | 27.201 | **<0.001** |
| **SARS-CoV-2 VOC**  Alpha/B.1.7.7  Pre-alpha** | 30.97  31.31 | 0.31  0.32 | 30.4-31.6  30.7-31.9 | 0.451  - | 0.796 | 0.451 |
| **SARS-CoV-2 lineage**  B.1.1.7  B.1.160  B.1.177  B.1.221 | 30.97  31.69  31.15  31.44 | 0.31  0.87  0.45  0.66 | 30.4-31.6  30.0-33.4  30.3-32.0  30.1-32.7 | -  0.437  0.750  0.521 | 0.298 | 0.827 |

Linear mixed models were used to assess changes in Ct values over time in feces. Estimated marginal means (EMM) were calculated with corresponding standard errors (SE) and 95% confidence intervals (CI). * *p*-value comparison with baseline (T0) ** Comprises pre-alpha lineages, including B.1.160, B.1.177, B.1.221, B.1, B.1.1.1, B.190, B.1.258, B.1.22, B.1.275, and B.1.356.

**Supplementary Table S4. Results of linear mixed models of Ct values in nasopharyngeal/throat (NPT)**

|  | **Estimated marginal means (EMM)** | **SE** | **95% CI** | ***p*-value*** | **F-statistic** | ***p*-value** |
| --- | --- | --- | --- | --- | --- | --- |
| **Timepoint**  T0 (Day 0)  T1 (Day 7)  T2 (Day 14)  T3 (Day 21)  T4 (Day 28)  T5 (Day 35)  T6 (Day 42) | 21.54  28.01  34.14  37.18  36.90  37.32  39.17 | 0.36  0.37  0.38  0.38  0.62  0.92  1.99 | 20.8-22.2  27.3-28.7  33.4-34.9  36.4-37.9  35.7-38.1  35.5-39.1  35.3-43.1 | -  **<0.001**  **<0.001 <0.001 <0.001**  **<0.001**  **<0.001** | 206.452 | **<0.001** |
| **Sex**  Female  Male | 31.20  30.36 | 0.37  0.43 | 30.5-31.9  29.5-31.2 | 0.140  - | 2.181 | 0.140 |
| **Age group (years)**  ≤15  16-50  >50 | 33.55  30.72  30.12 | 0.84  0.36  0.50 | 31.9-35.2  30.0-31.4  29.1-31.1 | -  **0.002**  **<0.001** | 6.287 | **0.002** |
| **SARS-CoV-2 VOC**  Alpha/B.1.1.7  Pre-alpha** | 29.82  31.17 | 0.44  0.43 | 28.9-30.7  30.3-32.0 | **0.029**  **-** | 4.817 | **0.029** |
| **SARS-CoV-2 lineage**  B.1.1.7  B.1.160  B.1.177  B.1.221 | 29.82  32.15  30.99  31.05 | 0.45  1.09  0.63  0.99 | 29.9-30.7  30.0-34.3  29.7-32.2  29.1-33.0 | -  **0.047**  0.131  0.256 | 1.855 | 0.136 |

Linear mixed models were used to assess changes in Ct values over time in NPT. Estimated marginal means (EMM) were calculated with corresponding standard errors (SE) and 95% confidence intervals (CI). * *p*-value comparison with baseline (T0) ** Comprises pre-alpha lineages, including B.1.160, B.1.177, B.1.221, B.1, B.1.1.1, B.190, B.1.258, B.1.22, B.1.275, and B.1.356.

**
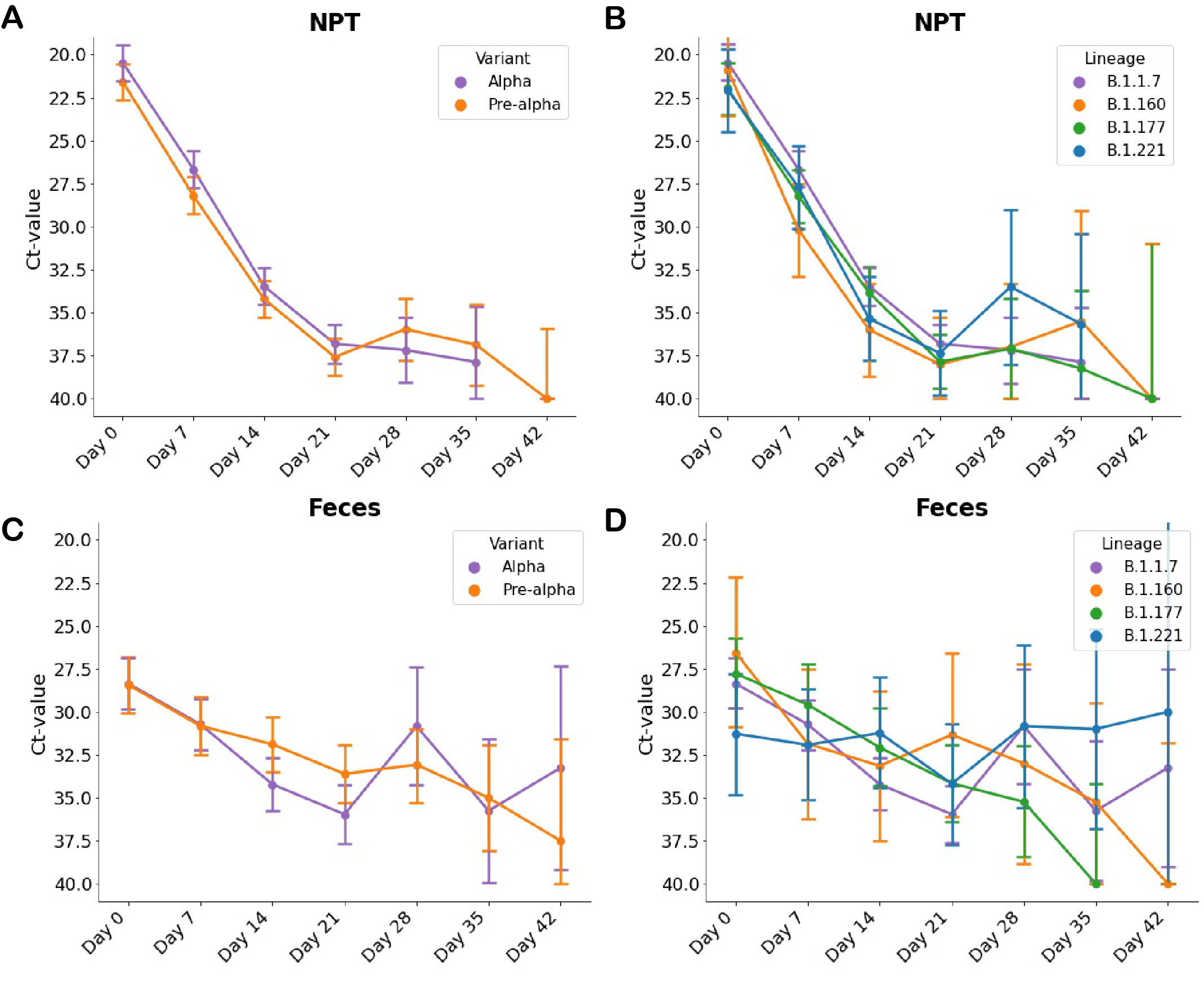
**

**Supplementary Figure S2. Temporal dynamics in Ct values in NPT and fecal samples by variant and lineage.** (A) NPT viral RNA shedding by variant alpha and pre-alpha. (B) NPT viral RNA shedding by lineage. (C) Fecal viral RNA shedding by variant alpha and pre-alpha. (D) Fecal viral RNA shedding by lineage. Data are presented as estimated marginal means (EMM) of Ct values. The error bars represent the 95% confidence intervals. Abbreviations: NPT, nasopharyngeal/throat.

**
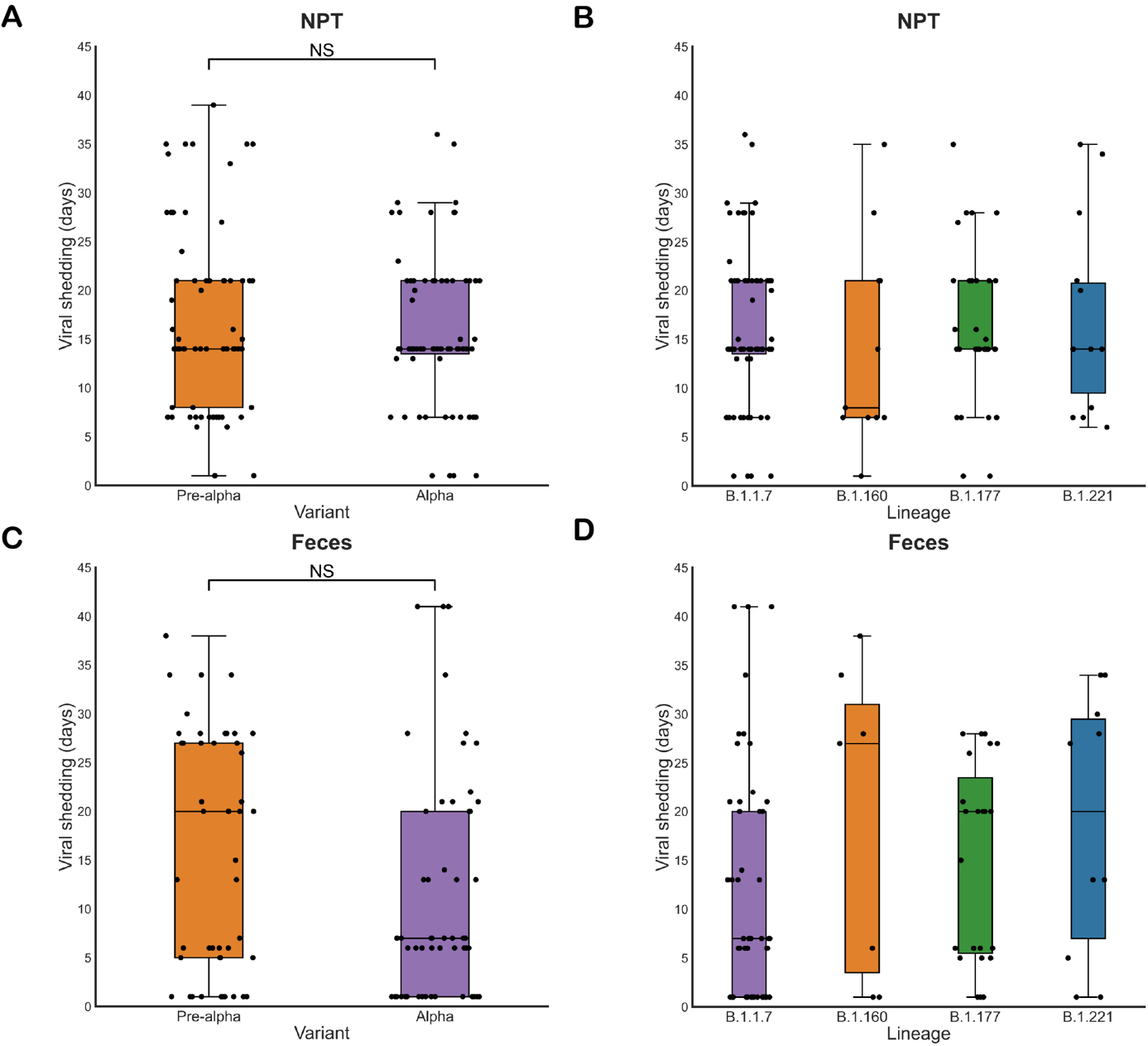
**

**Supplementary Figure S3. Duration of viral shedding (in days) in NPT and fecal samples by variant and lineage.** (A) NPT viral RNA shedding by variant (pre-alpha vs alpha) (*p*=0.953). (B) NPT viral RNA shedding by SARS-CoV-2 lineages (*p*=0.819). (C) Fecal viral RNA shedding by variant (pre-alpha vs. alpha) (*p*=0.106). (D) Fecal viral RNA shedding by SARS-CoV-2 lineages (*p*=0.281). Each point denotes the maximum duration of viral shedding per individual. Abbreviations: NPT, nasopharyngeal/throat; NS, non-significant.

**Supplementary Table S5. Univariable and multivariable logistic regression analyses of NPT long RNA shedding with clinical parameters.**

|  |  |  | **Univariable analysis** | | | **Multivariable analysis** | | |
| --- | --- | --- | --- | --- | --- | --- | --- | --- |
|  | ***n**** | **NPT long RNA shedding** | **OR** | **95% CI** | ***p*-value** | **OR** | **95% CI** | ***p*-value** |
| Total | 171 | 67 (39.2) |  |  |  |  |  |  |
| Age group |  |  |  |  | X^2^ test for trend, *p*=**0.006** |  |  |  |
| ≤ 15 | 21 | 4 (19.0) | - | - | - |  |  |  |
| 16-50 | 103 | 38 (36.9) | 2.49 | 0.78-7.93 | 0.115 |  |  |  |
| > 50 | 47 | 25 (53.2) | 4.83 | 1.41-16.53 | **0.009** |  |  |  |
| Sex |  |  |  |  |  |  |  |  |
| Female | 98 | 41 (41.8) | 1.30 | 0.70-2.43 | 0.410 |  |  |  |
| Male | 73 | 26 (35.6) | - | - | - |  |  |  |
| Smoking status |  |  |  |  |  |  |  |  |
| Current  *Yes*  *No* | 9  156 | 1 (11.1)  62 (39.7) | 0.19  - | 0.23-1.55  - | 0.121  - |  |  |  |
| Ever  *Yes*  *No* | 45  120 | 17 (37.8)  46 (38.3) | 0.98  - | 0.48-1.98  - | 0.948  - |  |  |  |
| Obesity  *Yes*  *No* | 17  112 | 4 (23.5)  44 (39.3) | 0.48  - | 0.15-1.55  - | 0.218  - |  |  |  |
| Variant |  |  |  |  |  |  |  |  |
| Pre-alpha | 72 | 27 (37.5) | - | - | - |  |  |  |
| Alpha | 69 | 28 (40.6) | 1.14 | 0.58-2.24 | 0.708 |  |  |  |
| Lineage |  |  |  |  | 0.475 |  |  |  |
| B.1.1.7 | 69 | 28 (40.6) | - | - | - |  |  |  |
| B.1.160 | 11 | 4 (36.4) | 0.84 | 0.22-3.13 | 0.791 |  |  |  |
| B.1.177 | 34 | 11 (32.4) | 0.70 | 0.30-1.66 | 0.419 |  |  |  |
| B.1.221 | 14 | 5 (35.7) | 0.81 | 0.25-2.69 | 0.735 |  |  |  |
| Number of comorbidities |  |  |  |  | X^2^ test for trend, *p*=0.832 |  |  |  |
| 0 | 116 | 45 (38.8) | - | - | - |  |  |  |
| 1 | 30 | 11 (36.7) | 0.91 | 0.40-2.10 | 0.831 |  |  |  |
| 2-5 | 21 | 9 (42.9) | 1.18 | 0.46-3.03 | 0.726 |  |  |  |
| Type of comorbidities |  |  |  |  |  |  |  |  |
| Cardiovascular  *Yes*  *No* | 14  152 | 6 (42.9)  58 (38.2) | 1.22  - | 0.40-3.68  - | 0.730  - |  |  |  |
| Pulmonary  *Yes*  *No* | 13  152 | 5 (38.5)  58 (38.2) | 1.01  - | 0.32-3.25  - | 0.983  - |  |  |  |
| Endocrine  *Yes*  *No* | 11  155 | 8 (72.7)  56 (36.1) | 4.71  - | 1.20-18.49  - | **0.026**  - | 4.29 | 1.06-17.45 | **0.042** |
| Rheumatolocial  *Yes*  *No* | 4  161 | 1 (25.0)  63 (39.1) | 0.52  - | 0.05-5.10  - | 0.573  - |  |  |  |
| Malignancy  *Yes*  *No* | 5  140 | 3 (60.0)  48 (34.3) | 2.88  - | 0.46-17.80  - | 0.256  - |  |  |  |
| Neurological  *Yes*  *No* | 4  142 | 2 (50.0)  49 (34.5) | 1.90  - | 0.26-13.89  - | 0.528  - |  |  |  |
| Hematological  *Yes*  *No* | 4  161 | 2 (50.0)  62 (38.5) | 1.60  - | 0.22-11.63  - | 0.644  - |  |  |  |
| Complaints |  |  |  |  |  |  |  |  |
| at 3 months  *Yes*  *No* | 54  64 | 20 (37.0)  22 (34.4) | 1.12  - | 0.53-2.39  - | 0.763  - |  |  |  |
| at 6 months  *Yes*  *No* | 49  73 | 18 (36.7)  25 (34.2) | 1.12  - | 0.52-2.37  - | 0.778  - |  |  |  |
| at 12 months  *Yes*  *No* | 34  87 | 10 (29.4)  32 (36.8) | 0.72  - | 0.30-1.69  - | 0.444  - |  |  |  |
| at 18 months  *Yes*  *No* | 19  61 | 6 (31.6)  22 (36.1) | 0.82  - | 0.27-2.46  - | 0.720  - |  |  |  |
| Type of symptoms |  |  |  |  |  |  |  |  |
| Dry cough |  |  |  |  |  |  |  |  |
| At presentation  *Yes*  *No* | 78  90 | 34 (43.6)  32 (35.6) | 1.40  - | 0.75-2.61  - | 0.483  - |  |  |  |
| Anytime  *Yes*  *No* | 110  61 | 47 (42.7)  20 (32.8) | 1.53  - | 0.80-2.94  - | 0.202  - |  |  |  |
| Sputum |  |  |  |  |  |  |  |  |
| At presentation  *Yes*  *No* | 41  127 | 20 (48.8)  46 (36.2) | 1.68  - | 0.82-3.42  - | 0.152  - |  |  |  |
| Anytime  *Yes*  *No* | 66  105 | 29 (43.9)  38 (36.2) | 1.38  - | 0.74-2.59  - | 0.312  - |  |  |  |
| Dyspnea |  |  |  |  |  |  |  |  |
| At presentation  *Yes*  *No* | 34  135 | 16 (47.1)  50 (37.0) | 1.51  - | 0.71-3.23  - | 0.284  - |  |  |  |
| Anytime  *Yes*  *No* | 65  106 | 30 (46.2)  37 (34.9) | 1.60  - | 0.85-3.00  - | 0.144  - |  |  |  |
| Fever |  |  |  |  |  |  |  |  |
| At presentation  *Yes*  *No* | 50  117 | 25 (50.0)  41 (35.0) | 1.85  - | 0.95-3.63  - | 0.070  - |  |  |  |
| Anytime  *Yes*  *No* | 63  108 | 32 (50.8)  35 (32.4) | 2.15  - | 1.14-4.07  - | **0.018**  - |  |  |  |
| Anosmia |  |  |  |  |  |  |  |  |
| At presentation  *Yes*  *No* | 49  118 | 20 (40.8)  47 (39.8) | 1.04  - | 0.53-2.05  - | 0.906  - |  |  |  |
| Anytime  *Yes*  *No* | 103  69 | 45 (43.7)  22 (32.4) | 1.62  - | 0.86-3.08  - | 0.137  - |  |  |  |
| Sore throat |  |  |  |  |  |  |  |  |
| At presentation  *Yes*  *No* | 67  101 | 28 (41.8)  39 (38.6) | 1.14  - | 0.61-2.14  - | 0.680  - |  |  |  |
| Anytime  *Yes*  *No* | 92  79 | 37 (40.2)  30 (38.0) | 1.10  - | 0.59-2.04  - | 0.765  - |  |  |  |
| Headache |  |  |  |  |  |  |  |  |
| At presentation  *Yes*  *No* | 94  75 | 41 (43.6)  26 (34.7) | 1.46  - | 0.78-2.73  - | 0.237  - |  |  |  |
| Anytime  *Yes*  *No* | 126  45 | 50 (39.7)  17 (37.8) | 1.08  - | 0.54-2.18  - | 0.822  - |  |  |  |
| Fatigue |  |  |  |  |  |  |  |  |
| At presentation  *Yes*  *No* | 82  85 | 36 (43.9)  28 (32.9) | 1.59  - | 0.85-2.99  - | 0.145  - |  |  |  |
| Anytime  *Yes*  *No* | 130  41 | 53 (40.8)  14 (34.1) | 1.33  - | 0.64-2.77  - | 0.449  - |  |  |  |
| Abdominal pain |  |  |  |  |  |  |  |  |
| At presentation  *Yes*  *No* | 20  149 | 9 (45.0)  57 (38.3) | 1.32  - | 0.52-3.38  - | 0.562  - |  |  |  |
| Anytime  *Yes*  *No* | 45  126 | 19 (42.2)  48 (38.1) | 1.19  - | 0.59-2.37  - | 0.626  - |  |  |  |
| Diarrhea |  |  |  |  |  |  |  |  |
| At presentation  *Yes*  *No* | 22  147 | 13 (59.1)  54 (36.7) | 2.49  - | 1.00-6.20  - | **0.046**  - |  |  |  |
| Anytime  *Yes*  *No* | 42  129 | 20 (47.6)  47 (36.4) | 1.59  - | 0.79-3.21  - | 0.197  - |  |  |  |
| Anorexia |  |  |  |  |  |  |  |  |
| At presentation  *Yes*  *No* | 31  138 | 12 (38.7)  54 (39.1) | 0.98  - | 0.44-2.19  - | 0.965  - |  |  |  |
| Anytime  *Yes*  *No* | 62  109 | 31 (50.0)  36 (33.0) | 2.03  - | 1.07-3.84  - | **0.029**  - |  |  |  |
| Vomiting |  |  |  |  |  |  |  |  |
| At presentation  *Yes*  *No* | 11  158 | 4 (36.4)  62 (39.2) | 0.89  - | 0.25-3.15  - | 0.850  - |  |  |  |
| Anytime  *Yes*  *No* | 38  133 | 14 (36.8)  53 (39.8) | 0.88  - | 0.42-1.86  - | 0.738  - |  |  |  |
| Rhinorrhea |  |  |  |  |  |  |  |  |
| At presentation  *Yes*  *No* | 58  110 | 29 (50.0)  38 (34.5) | 1.90  - | 0.99-3.62  - | 0.052  - |  |  |  |
| Anytime  *Yes*  *No* | 101  70 | 40 (39.6)  27 (38.6) | 1.04  - | 0.56-1.95  - | 0.892  - |  |  |  |
| Chills |  |  |  |  |  |  |  |  |
| At presentation  *Yes*  *No* | 43  127 | 24 (55.8)  43 (33.9) | 2.47  - | 1.22-5.00  - | **0.011**  - |  |  |  |
| Anytime  *Yes*  *No* | 64  107 | 35 (54.7)  32 (29.9) | 2.83  - | 1.49-5.38  - | **0.001**  - | 2.76 | 1.42-5.35 | **0.003** |
| Myalgia |  |  |  |  |  |  |  |  |
| At presentation  *Yes*  *No* | 69  100 | 33 (47.8)  34 (34.0) | 1.78  - | 0.95-3.33  - | 0.071  - |  |  |  |
| Anytime  *Yes*  *No* | 93  78 | 40 (43.0)  27 (34.6) | 1.43  - | 0.77-2.65  - | 0.263  - |  |  |  |
| Arthralgia |  |  |  |  |  |  |  |  |
| At presentation  *Yes*  *No* | 17  151 | 10 (58.8)  56 (37.1) | 2.42  - | 0.87-6.73  - | 0.082  - |  |  |  |
| Anytime  *Yes*  *No* | 35  136 | 15 (42.9)  52 (38.2) | 1.21  - | 0.57-2.57  - | 0.617  - |  |  |  |

***** Represents the number of positive individuals of a certain variable, among all individuals with available data on NPT long RNA shedding (*n*=177). Abbreviations: CI, confidence interval; OR, odds ratio.

**Supplementary Table S6. Univariable and multivariable logistic regression analyses of fecal long RNA shedding with clinical parameters.**

|  |  |  | **Univariable analysis** | | | **Multivariable analysis** | | |
| --- | --- | --- | --- | --- | --- | --- | --- | --- |
|  | ***n**** | **Fecal long RNA shedding** | **OR** | **95% CI** | ***p*-value** | **OR** | **95% CI** | **p-value** |
| Total | 132 | 53 (40.2) |  |  |  |  |  |  |
| Age group |  |  |  |  | X^2^ test for trend, p=**0.030** |  |  |  |
| ≤ 15 | 12 | 10 (83.3) | - | - | - |  |  |  |
| 16-50 | 80 | 29 (36.3) | 0.11 | 0.02-0.56 | **0.002** | 0.12 | 0.02-0.67 | **0.015** |
| > 50 | 40 | 14 (35.0) | 0.11 | 0.02-0.56 | **0.003** | 0.09 | 0.02-0.55 | **0.009** |
| Sex |  |  |  |  |  |  |  |  |
| Female | 78 | 26 (33.3) | 0.50 | 0.25-1.02 | 0.055 | 0.39 | 0.17-0.89 | **0.025** |
| Male | 54 | 27 (50.0) | - | - | - |  |  |  |
| Smoking status |  |  |  |  |  |  |  |  |
| Current  *Yes*  *No* | 5  125 | 0 (0.0)  51 (40.8) | NA**  - | NA**  - | NA**  - |  |  |  |
| Ever  *Yes*  *No* | 34  96 | 10 (29.4)  41 (42.7) | 0.56  - | 0.24-1.30  - | 0.175  - |  |  |  |
| Obesity  *Yes*  *No* | 17  94 | 6 (35.3)  34 (36.2) | 0.96  - | 0.33-2.84  - | 0.945  - |  |  |  |
| Variant |  |  |  |  |  |  |  |  |
| Pre-alpha | 51 | 25 (49.0) | - | - | - |  |  |  |
| Alpha | 58 | 17 (29.3) | 0.43 | 0.20-0.95 | **0.036** |  |  |  |
| Lineage |  |  |  |  | 0.092 |  |  |  |
| B.1.1.7 | 58 | 17 (29.3) | - | - | - |  |  |  |
| B.1.160 | 7 | 4 (57.1) | 3.22 | 0.65-15.93 | 0.153 |  |  |  |
| B.1.177 | 25 | 13 (52.0) | 2.61 | 0.99-6.87 | 0.052 |  |  |  |
| B.1.221 | 12 | 5 (41.7) | 1.72 | 0.48-6.19 | 0.405 |  |  |  |
| Number of comorbidities |  |  |  |  | X^2^ test for trend, p=0.161 |  |  |  |
| 0 | 89 | 42 (47.2) | - | - | - |  |  |  |
| 1 | 25 | 4 (16.0) | 0.21 | 0.07-0.67 | **0.008** |  |  |  |
| 2-5 | 17 | 6 (35.3) | 0.61 | 0.21-1.79 | 0.370 |  |  |  |
| Type of comorbidities |  |  |  |  |  |  |  |  |
| Cardiovascular  *Yes*  *No* | 12  118 | 2 (16.7)  49 (41.5) | 0.28  - | 0.06-1.34  - | 0.112  - |  |  |  |
| Pulmonary  *Yes*  *No* | 13  116 | 5 (38.5)  45 (38.8) | 0.99  - | 0.30-3.20  - | 0.981  - |  |  |  |
| Endocrine  *Yes*  *No* | 8  122 | 1 (12.5)  50 (41.0) | 0.21  - | 0.03-1.72  - | 0.145  - |  |  |  |
| Rheumatolocial  *Yes*  *No* | 3  126 | 0 (0.0)  51 (40.5) | NA***  - | NA***  - | NA***  - |  |  |  |
| Malignancy  *Yes*  *No* | 4  117 | 2 (50.0)  44 (37.6) | 1.66  - | 0.23-12.20  - | 0.619  - |  |  |  |
| Neurological  *Yes*  *No* | 4  119 | 2 (50.0)  45 (37.8) | 1.64  - | 0.22-12.09  - | 0.625  - |  |  |  |
| Hematological  *Yes*  *No* | 4  125 | 3 (75.0)  48 (38.4) | 4.81  - | 0.49-47.60  - | 0.179  - |  |  |  |
| Complaints |  |  |  |  |  |  |  |  |
| at 3 months  *Yes*  *No* | 46  54 | 14 (30.4)  20 (37.0) | 0.74  - | 0.32-1.72  - | 0.487  - |  |  |  |
| at 6 months  *Yes*  *No* | 43  63 | 13 (30.2)  25 (39.7) | 0.66  - | 0.29-1.50  - | 0.319  - |  |  |  |
| at 12 months  *Yes*  *No* | 30  69 | 8 (26.7)  30 (43.5) | 0.47  - | 0.19-1.21  - | 0.114  - |  |  |  |
| at 18 months  *Yes*  *No* | 18  52 | 8 (44.4)  22 (42.3) | 1.09  - | 0.37-3.21  - | 0.875  - |  |  |  |
| Type of symptoms |  |  |  |  |  |  |  |  |
| Dry cough |  |  |  |  |  |  |  |  |
| At presentation  *Yes*  *No* | 66  64 | 29 (43.9)  24 (37.5) | 1.31  - | 0.65-2.64  - | 0.455  - |  |  |  |
| Anytime  *Yes*  *No* | 93  39 | 33 (35.5)  20 (51.3) | 0.52  - | 0.25-1.12  - | 0.091  - |  |  |  |
| Sputum |  |  |  |  |  |  |  |  |
| At presentation  *Yes*  *No* | 39  92 | 12 (30.8)  41 (44.6) | 0.55  - | 0.25-1.22  - | 0.141  - |  |  |  |
| Anytime  *Yes*  *No* | 60  72 | 20 (33.3)  33 (45.8) | 0.59  - | 0.29-1.20  - | 0.145  - |  |  |  |
| Dyspnea |  |  |  |  |  |  |  |  |
| At presentation  *Yes*  *No* | 27  104 | 9 (33.3)  44 (42.3) | 0.68  - | 0.28-1.66  - | 0.397  - |  |  |  |
| Anytime  *Yes*  *No* | 50  82 | 16 (32.0)  37 (45.1) | 0.57  - | 0.27-1.20  - | 0.136  - |  |  |  |
| Fever |  |  |  |  |  |  |  |  |
| At presentation  *Yes*  *No* | 43  86 | 13 (30.2)  39 (45.3) | 0.52  - | 0.24-1.14  - | 0.099  - |  |  |  |
| Anytime  *Yes*  *No* | 53  79 | 17 (32.1)  36 (45.6) | 0.56  - | 0.27-1.17  - | 0.121  - |  |  |  |
| Anosmia |  |  |  |  |  |  |  |  |
| At presentation  *Yes*  *No* | 41  87 | 15 (36.6)  38 (43.7) | 0.74  - | 0.35-1.60  - | 0.447  - |  |  |  |
| Anytime  *Yes*  *No* | 84  48 | 30 (35.7)  23 (47.9) | 0.60  - | 0.29-1.24  - | 0.169  - |  |  |  |
| Sore throat |  |  |  |  |  |  |  |  |
| At presentation  *Yes*  *No* | 54  75 | 21 (38.9)  32 (42.7) | 0.89  - | 0.42-1.75  - | 0.667  - |  |  |  |
| Anytime  *Yes*  *No* | 74  58 | 28 (37.8)  25 (43.1) | 0.80  - | 0.40-1.62  - | 0.540  - |  |  |  |
| Headache |  |  |  |  |  |  |  |  |
| At presentation  *Yes*  *No* | 78  53 | 27 (34.6)  26 (49.1) | 0.55  - | 0.27-1.12  - | 0.098  - |  |  |  |
| Anytime  *Yes*  *No* | 101  31 | 36 (35.6)  17 (54.8) | 0.46  - | 0.20-1.03  - | 0.057  - |  |  |  |
| Fatigue |  |  |  |  |  |  |  |  |
| At presentation  *Yes*  *No* | 67  62 | 26 (38.8)  25 (40.3) | 0.94  - | 0.46-1.90  - | 0.860  - |  |  |  |
| Anytime  *Yes*  *No* | 103  29 | 34 (33.0)  19 (65.5) | 0.26  - | 0.11-0.62  - | **0.002**  - | 0.29 | 0.11-0.76 | **0.011** |
| Abdominal pain |  |  |  |  |  |  |  |  |
| At presentation  *Yes*  *No* | 16  114 | 5 (31.3)  48 (42.1) | 0.63  - | 0.20-1.92  - | 0.408  - |  |  |  |
| Anytime  *Yes*  *No* | 36  96 | 11 (30.6)  42 (43.8) | 0.57  - | 0.25-1.28  - | 0.168  - |  |  |  |
| Diarrhea |  |  |  |  |  |  |  |  |
| At presentation  *Yes*  *No* | 18  113 | 7 (38.9)  46 (40.7) | 0.93  - | 0.33-2.57  - | 0.884  - |  |  |  |
| Anytime  *Yes*  *No* | 36  96 | 11 (30.6)  42 (43.8) | 0.57  - | 0.25-1.28  - | 0.168  - |  |  |  |
| Anorexia |  |  |  |  |  |  |  |  |
| At presentation  *Yes*  *No* | 26  105 | 7 (26.9)  46 (43.8) | 0.47  - | 0.18-1.22  - | 0.116  - |  |  |  |
| Anytime  *Yes*  *No* | 49  83 | 16 (32.7)  37 (44.6) | 0.60  - | 0.29-1.26  - | 0.177  - |  |  |  |
| Vomiting |  |  |  |  |  |  |  |  |
| At presentation  *Yes*  *No* | 12  119 | 8 (66.7)  44 (37.0) | 3.41  - | 0.97-11.98  - | 0.056 | 5.31 | 1.29-21.79 | **0.020** |
| Anytime  *Yes*  *No* | 36  96 | 13 (36.1)  40 (41.7) | 0.79  - | 0.36-1.75  - | 0.562  - |  |  |  |
| Rhinorrhea |  |  |  |  |  |  |  |  |
| At presentation  *Yes*  *No* | 45  85 | 21 (46.7)  32 (37.6) | 1.45  - | 0.70-3.01  - | 0.319  - |  |  |  |
| Anytime  *Yes*  *No* | 80  52 | 33 (41.3)  20 (38.5) | 1.12  - | 0.55-2.30  - | 0.749  - |  |  |  |
| Chills |  |  |  |  |  |  |  |  |
| At presentation  *Yes*  *No* | 35  97 | 11 (31.4)  42 (43.3) | 0.60  - | 0.27-1.36  - | 0.219  - |  |  |  |
| Anytime  *Yes*  *No* | 54  78 | 17 (31.5)  36 (46.2) | 0.54  - | 0.26-1.11  - | 0.091  - |  |  |  |
| Myalgia |  |  |  |  |  |  |  |  |
| At presentation  *Yes*  *No* | 57  73 | 19 (33.3)  34 (46.6) | 0.57  - | 0.28-1.18  - | 0.127  - |  |  |  |
| Anytime  *Yes*  *No* | 76  56 | 24 (31.6)  29 (51.8) | 0.43  - | 0.21-0.88  - | **0.019**  - |  |  |  |
| Arthralgia |  |  |  |  |  |  |  |  |
| At presentation  *Yes*  *No* | 12  118 | 5 (41.7)  48 (40.7) | 1.04  - | 0.31-3.48  - | 0.947  - |  |  |  |
| Anytime  *Yes*  *No* | 26  106 | 7 (26.9)  46 (43.3) | 0.48  - | 0.19-1.24  - | 0.125  - |  |  |  |

***** Represents the number of positive individuals of a certain variable, among all individuals with available data on fecal long RNA shedding (*n*=132). **/*** Since none of the long shedders were current smokers (**) or had rheumatological disease (***), no reliable odds ratio could be calculated. Abbreviations: CI, confidence interval; OR, odds ratio.

**Supplementary Table S7. Results of linear mixed models of log-transformed anti-S1 SARS-CoV-2 IgG titers in feces.**

|  | **Estimated marginal means (EMM)** | **SE** | **95% CI** | ***p*-value*** | **F-statistic** | ***p*-value** |
| --- | --- | --- | --- | --- | --- | --- |
| **Timepoint**  21 days  3 months  6 months  12 months  18 months | 2.05  2.45  2.68  2.95  3.02 | 0.06  0.06  0.06  0.06  0.07 | 1.93-2.16  2.33-2.57  2.56-2.80  2.83-3.07  2.88-3.15 | -  **<0.001**  **<0.001**  **<0.001**  **<0.001** | 40.522 | **<0.001** |
| **Long RNA shedder**  Yes  No | 2.71  2.54 | 0.05  0.04 | 2.62-2.81  2.46-2.62 | **0.006**  - | 7.537 | **0.006** |

Linear mixed models were used to assess changes in log-transformed anti-S1 SARS-CoV-2 IgG titers over long-term in feces. Estimated marginal means (EMM) were calculated with corresponding standard errors (SE) and 95% confidence intervals (CI). Abbreviations: S1, spike. * *p*-value comparison with baseline (T0)

**Supplementary Table S8. Results of linear mixed models of log-transformed anti-S1 SARS-CoV-2 IgG titers in NPT.**

|  | **Estimated marginal means (EMM)** | **SE** | **95% CI** | ***p*-value*** | **F-statistic** | ***p*-value** |
| --- | --- | --- | --- | --- | --- | --- |
| **Timepoint**  21 days  3 months  6 months  12 months  18 months | 2.05  2.41  2.62  2.90  3.00 | 0.06  0.06  0.06  0.06  0.06 | 1.93-2.16  2.29-2.52  2.50-2.73  2.79-3.02  2.88-3.13 | -  **<0.001**  **<0.001**  **<0.001**  **<0.001** | 42.030 | **<0.001** |
| **Long RNA shedder**  Yes  No | 2.65  2.53 | 0.05  0.04 | 2.55-2.74  2.45-2.61 | 0.056  - | 3.658 | 0.056 |

Linear mixed models were used to assess changes in log-transformed anti-S1 SARS-CoV-2 IgG titers over long-term in NPT. Estimated marginal means (EMM) were calculated with corresponding standard errors (SE) and 95% confidence intervals (CI). Abbreviations: S1, spike. * *p*-value comparison with baseline (T0).
