# Supplementary figures and images for: Temporal Dynamics and (Para)Clinical Factors Associated With (Long) Viral RNA Shedding in COVID‐19 Nonhospitalized Individuals – The COVID‐HOME Study
Source: J Med Virol. 2024 Dec 17;96(12):e70125. doi: 10.1002/jmv.70125 (PMC11653057; doi:10.1002/jmv.70125)

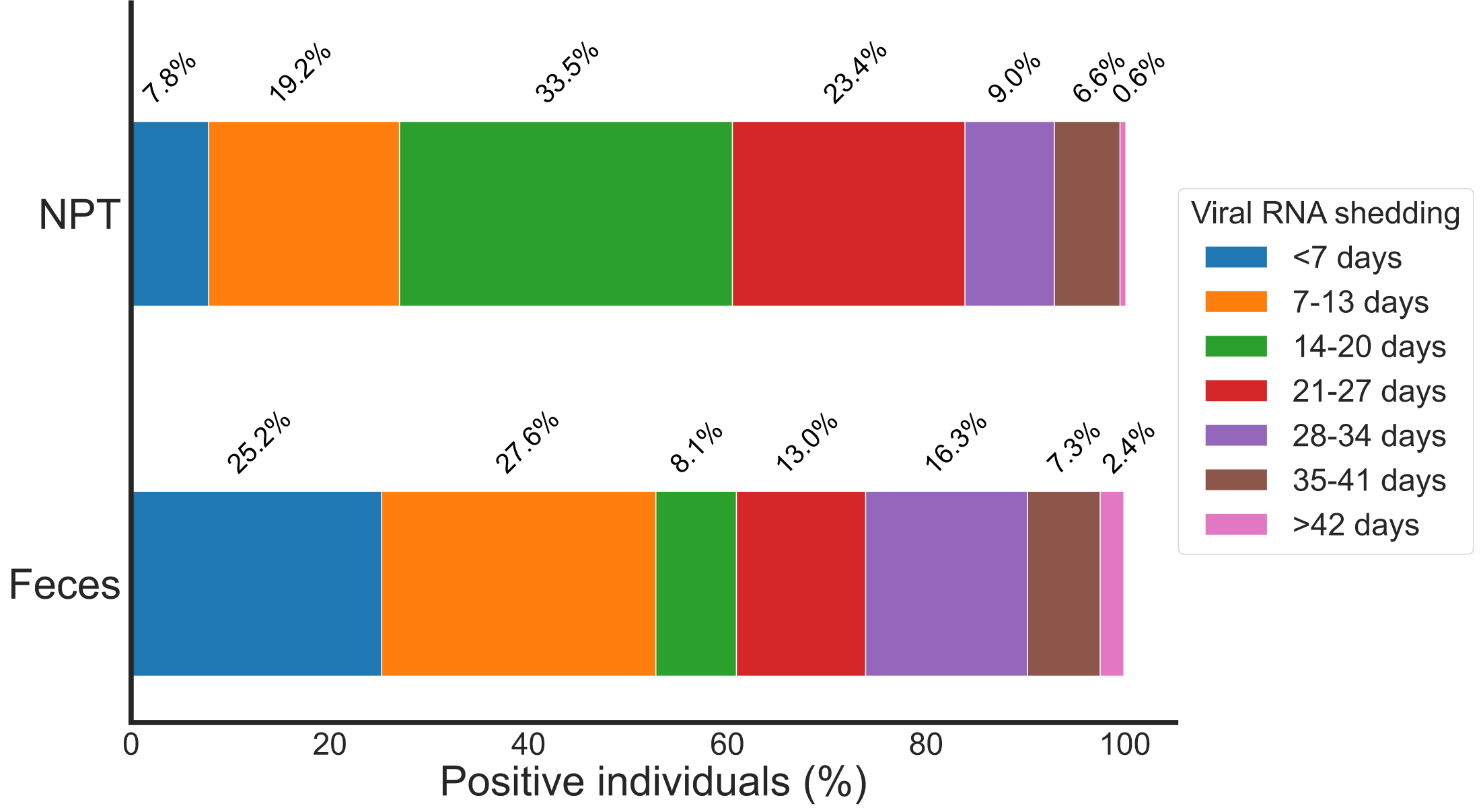

Supplement: Supplementary file 2 — Supporting information. [file JMV-96-e70125-s002.tif]

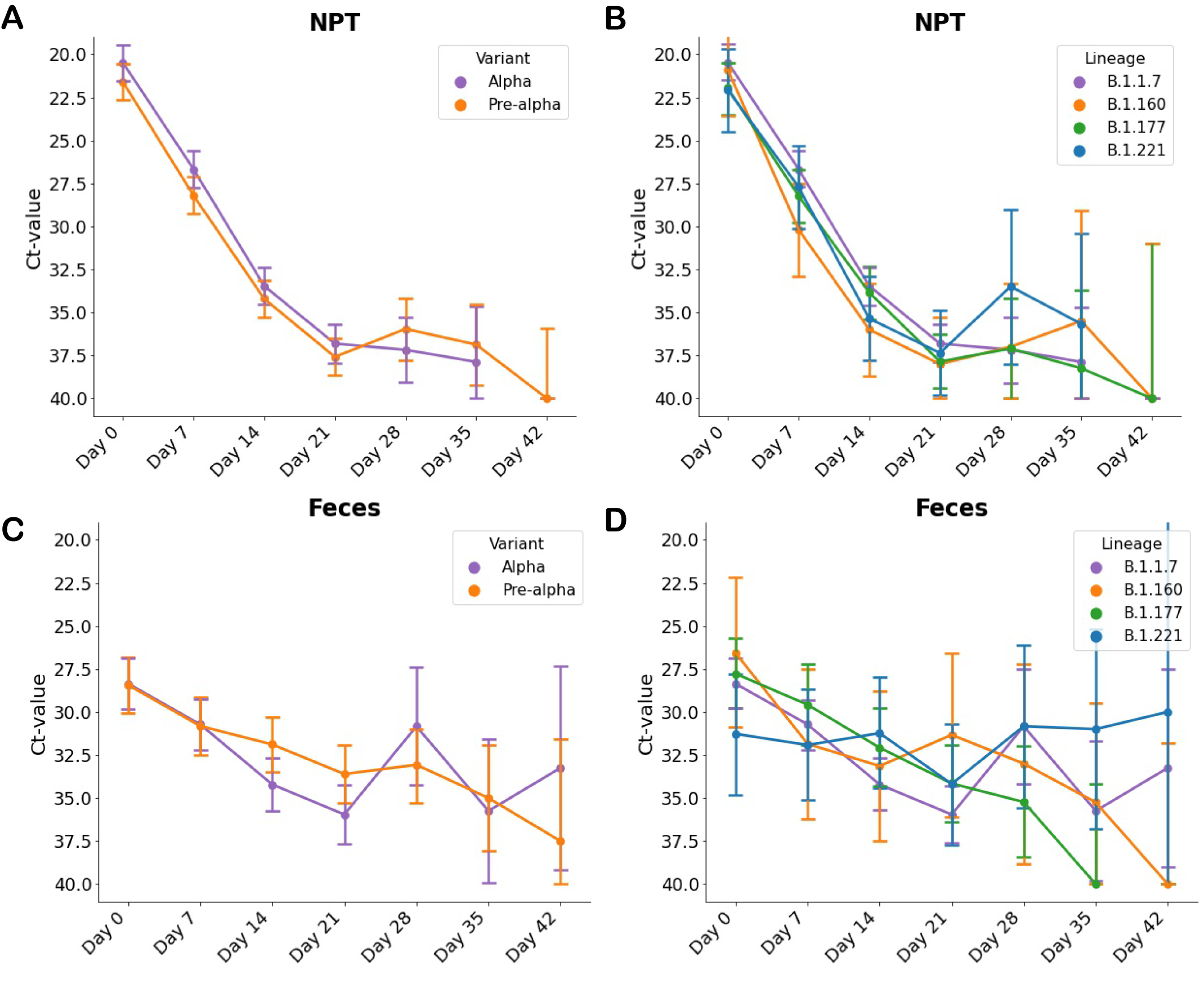

Supplement: Supplementary file 3 — Supporting information. [file JMV-96-e70125-s001.tif]

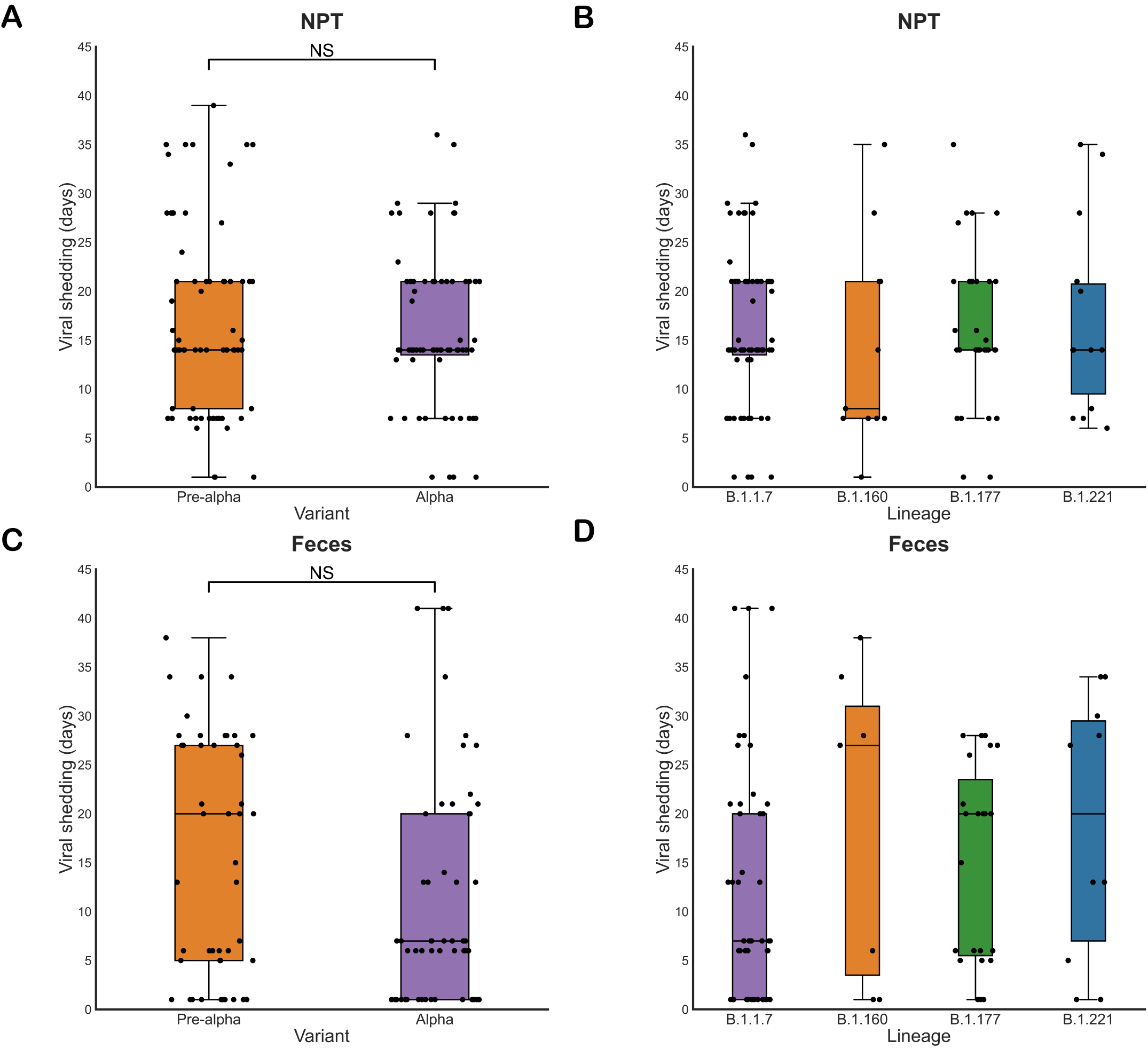

Supplement: Supplementary file 4 — Supporting information. [file JMV-96-e70125-s003.tif]
